# Critical Role of Transient Activity of MT1-MMP for ECM Degradation in Invadopodia

**DOI:** 10.1371/journal.pcbi.1003086

**Published:** 2013-05-30

**Authors:** Ayako Watanabe, Daisuke Hosino, Naohiko Koshikawa, Motoharu Seiki, Takashi Suzuki, Kazuhisa Ichikawa

**Affiliations:** 1 Division of Mathematical Oncology, The Institute of Medical Science, The University of Tokyo, Minato-ku, Tokyo, Japan; 2 Division of Cancer Cell Research, The Institute of Medical Science, The University of Tokyo, Minato-ku, Tokyo, Japan; 3 JST, CREST, Chiyoda-ku, Tokyo, Japan; 4 Division of Mathematical Science, Graduate School of Engineering Science, Osaka University, Toyonaka, Osaka, Japan; University of Virginia, United States of America

## Abstract

Focal degradation of extracellular matrix (ECM) is the first step in the invasion of cancer cells. MT1-MMP is a potent membrane proteinase employed by aggressive cancer cells. In our previous study, we reported that MT1-MMP was preferentially located at membrane protrusions called invadopodia, where MT1-MMP underwent quick turnover. Our computer simulation and experiments showed that this quick turnover was essential for the degradation of ECM at invadopodia (Hoshino, D., et al., (2012) PLoS Comp. Biol., 8: e1002479). Here we report on characterization and analysis of the ECM-degrading activity of MT1-MMP, aiming at elucidating a possible reason for its repetitive insertion in the ECM degradation. First, in our computational model, we found a very narrow transient peak in the activity of MT1-MMP followed by steady state activity. This transient activity was due to the inhibition by TIMP-2, and the steady state activity of MT1-MMP decreased dramatically at higher TIMP-2 concentrations. Second, we evaluated the role of the narrow transient activity in the ECM degradation. When the transient activity was forcibly suppressed in computer simulations, the ECM degradation was heavily suppressed, indicating the essential role of this transient peak in the ECM degradation. Third, we compared continuous and pulsatile turnover of MT1-MMP in the ECM degradation at invadopodia. The pulsatile insertion showed basically consistent results with the continuous insertion in the ECM degradation, and the ECM degrading efficacy depended heavily on the transient activity of MT1-MMP in both models. Unexpectedly, however, low-frequency/high-concentration insertion of MT1-MMP was more effective in ECM degradation than high-frequency/low-concentration pulsatile insertion even if the time-averaged amount of inserted MT1-MMP was the same. The present analysis and characterization of ECM degradation by MT1-MMP together with our previous report indicate a dynamic nature of MT1-MMP at invadopodia and the importance of its transient peak in the degradation of the ECM.

## Author Summary

Metastasis is the major cause of death in cancer patients. If metastasis is blocked, the survival rate will be greatly increased. Cancer cells are surrounded by ECM (extracellular matrix), which prevents their free movement. MT1-MMP is a potent membrane proteinase that degrades ECM, which is the first step of cancer cell invasion. Thus, the control of MT1-MMP activity is a key to the prevention of metastasis. Here we found a sharp transient peak in the ECM-degrading activity of MT1-MMP by computer simulations, and computational elimination of this peak greatly prolonged the ECM degradation. MT1-MMP is transported intracellularly to the surface of the membrane of cancer cells, and its insertion into the membrane is thought to occur in a pulsatile manner. Therefore, we asked whether the ECM-degrading efficacy was the same in low-frequency/high-concentration and high-frequency/low-concentration insertions of MT1-MMP. Unexpectedly, the low-frequency/high-concentration regimen resulted in much faster ECM degradation even if the time-averaged amount of MT1-MMP insertion was the same. Thus, reduction of the sharp transient activity and vesicular content of MT1-MMP are important therapeutic targets.

## Introduction

Metastasis is the major cause of death in cancer patients. If metastasis is blocked, 90% of patients will survive [1]. Thus hindering metastasis should be a main therapeutic target. The first step of invasion is the focal degradation of extracellular matrix (ECM) surrounding cancer cells, and MT1-MMP, a membrane metalloproteinase, plays a critical role in this process [2]–[4]. Invadopodia, which are tiny protrusions found on the surface of malignant cancer cells, are the machinery involved in the focal degradation of ECM, and this is where MT1-MMP is accumulating [5]–[8], and causing degradation of ECM [5]. Thus, the activity of MT1-MMP at invadopodia is critical for ECM degradation and cancer cell invasion and metastasis.

MT1-MMP is blocked by an endogenous soluble inhibitor TIMP-2. On the other hand, TIMP-2 binds a proform of an endogenous soluble metalloproteinase MMP-2 (proMMP-2) forming a ternary complex of MT1-MMP.TIMP-2.proMMP-2 [9]. If additional MT1-MMP binds to the ternary complex through the hemopexin domain of MT1-MMP a quaternary complex is formed of MT1-MMP.MT1-MMP.TIMP-2.proMMP-2; proMMP-2 in the complex is processed by the newly bound TIMP-2-free MT1-MMP, which results in the release of active MMP-2 [10], [11]. Active MMP-2 degrades ECM together with MT1-MMP [12]–[16]. Thus, TIMP-2 plays dual roles as an MT1-MMP inhibitor and as an adaptor for MMP-2 activation, and thus acts as both inhibitor and enhancer of ECM degradation [9], [17]–[20]. In spite of these possibilities, a high TIMP-2 concentration leads to almost complete inhibition of MT1-MMP.

In order to overcome this inhibition, it is essential to recycle and replenish TIMP-free MT1-MMP to the membrane surface by vesicular trafficking whatever the route is. Thus the vesicular trafficking of MT1-MMP plays a more dynamic role in ECM degradation than simply transporting newly synthesized MT1-MMP to the membrane surface. In fact, pharmacological blockade of vesicle trafficking dramatically reduced the focal degradation of ECM at invadopodia [21]–[28].

However, the activation and deactivation mechanisms of MT1-MMP are not as simple as suggested in the previous report because various complexes can form from MT1-MMP, TIMP-2 and MMP-2 [23]. We suggested that these complexes play a role in the control of ECM degrading activity, and that the dynamic nature of these complexes is critical for the ECM degradation. However, the dynamics of the ECM-degrading activity of MT1-MMP still remains to be elucidated.

Here we analyze the activity of MT1-MMP in our computational model, which also was used in the previous report, with the aim of finding a possible reason for the repetitive insertion required for the ECM degradation [23]. We have found a very narrow transient activity of MT1-MMP with a half-width of less than 5 sec followed by a steady state activity. The transient activity depends on the TIMP-2 concentration, and is prominent at higher TIMP-2 concentrations. Importantly, we have found that the transient activity is crucial for the degradation of ECM, because in the absence of the transient activity, ECM degradation by MT1-MMP was heavily reduced. Finally, we test the effect of pulsatile insertion of MT1-MMP, which is a simulated vesicular insertion of MT1-MMP [26], [29]. The simulation shows a faster ECM degradation by insertion of a larger MT1-MMP content in a single vesicle, while the time-averaged amount of insertion is kept constant.

## Results

### ECM degradation is reduced by inhibiting vesicular trafficking of MT1-MMP

**Figure 1:**
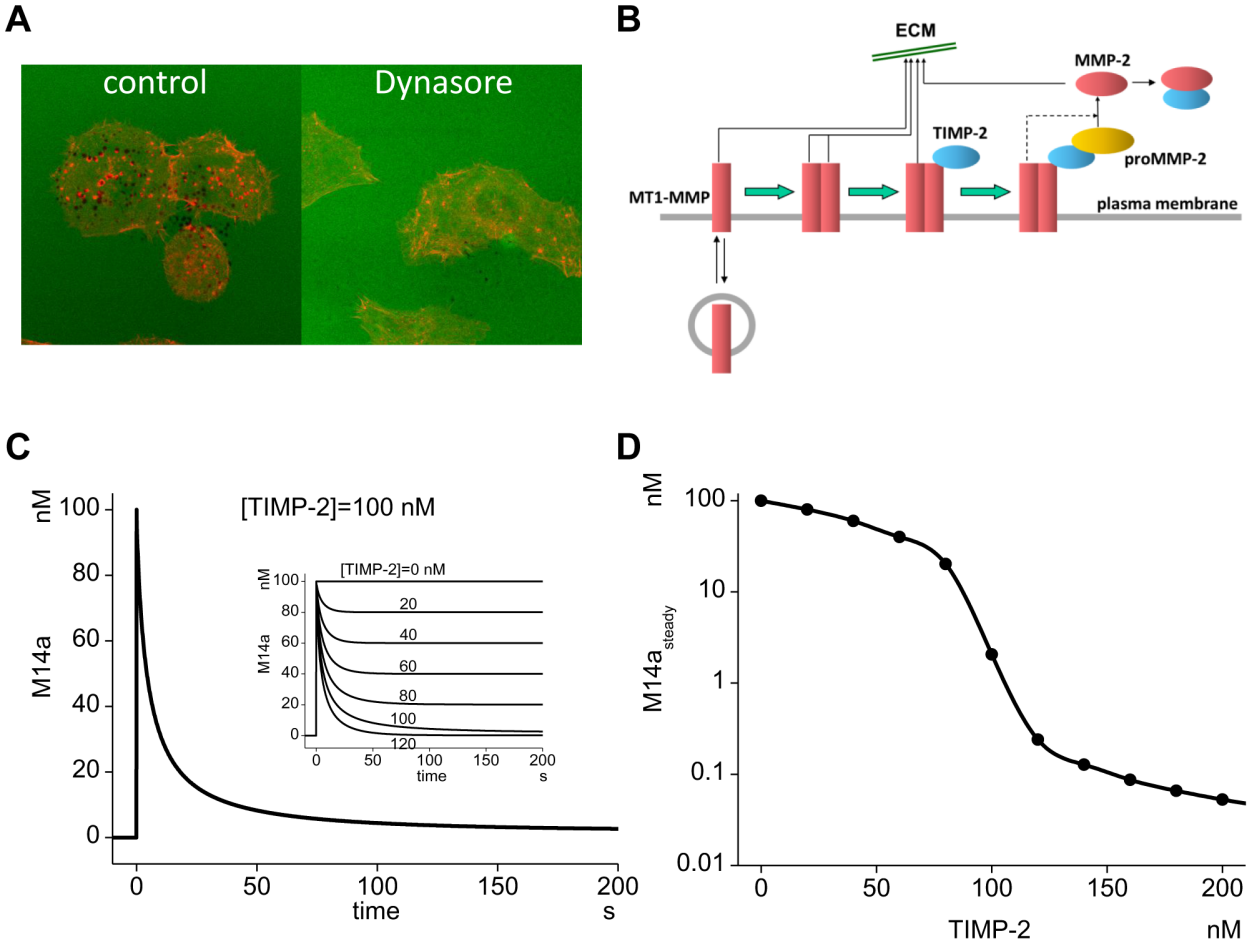
Critical role of the MT1-MMP turnover at invadopodia and a sharp transient peak in MT1-MMP. (A) Degradation of ECM by MT1-MMP is inhibited by the application of Dynasore blocking vesicular turnover. Green: ECM (fibronectin), Red: actin, Black dots: focal degradation of ECM. (B) Model used in the present study is schematically shown. MT1-MMP in the invadopodial membrane is inserted and internalized by vesicular trafficking, MT1-MMP forms dimer, ternary complex with TIMP-2 and proMMP-2. ProMMP-2 is activated by TIMP-2-free MT1-MMP. ECM is degraded by active complexes of MT1-MMP and MMP-2. (C) Simulation revealed the existence of a sharp transient peak at the initial phase of the formation of active MT1-MMP complexes (M14a). The height of the transient peak depends on the TIMP-2 concentration (inset). (D) Steady state level of M14a (M14a_steady_) is plotted as a function of TIMP-2 concentration. There is an abrupt decrease in M14a_steady_ in the region of TIMP-2 from 80 nM to 120 nM.

When head and neck squamous cell carcinoma SCC61 cells are cultured on glass plates coated with Dylight 633-labeled fibronectin over cross-linked gelatin, the loci of ECM degradation are visualized as dark spots (left panel in Figure 1A). ECM degradation in our system depends on MT1-MMP and its vesicular trafficking to the surface of invadopodia, since knockdown of MT1-MMP or the inhibition of vesicular transport reduces the number of ECM-degradation spots (right panel) [23].

In the previous report, we have shown that the turnover of MT1-MMP is required for the effective ECM degradation [23]. However, it is not known why the turnover is required for the ECM degradation. Since MT1-MMP is inhibited by the endogenous inhibitor TIMP-2, newly inserted MT1-MMP can readily be inhibited by TIMP-2. Therefore, a repetitive insertion of MT1-MMP may be required to overcome this inhibitory effect of TIMP-2 and provide an effective ECM degradation. In order to investigate this possibility, we ran a series of computer simulations, and analyzed the dynamic activity of MT1-MMP, which can be too fast to observe experimentally.

### A sharp transient activity of MT1-MMP is seen just after the insertion

The computational model is shown schematically in Figure 1B [23]. All possible complexes and their state transitions are shown in Figure S1, and an A-Cell model is shown in Figure S2, where MT1-MMP, TIMP-2, and MMP-2 are named M14, T2, and M2, respectively. In this model, schemes of the ECM degradation are not involved.

First we investigated the time course of the concentration of active complexes of MT1-MMP that play a role in ECM degradation. We defined M14a, which is the sum of the concentrations of M14, M14.M14, M14.M14.T2, and M14.M14.T2.M2, as a measure for the ECM degrading activity of MT1-MMP. The dimer M14.M14 was assumed to possess twice the activity of that of other complexes. We assumed a surface expression of 100 nM MT1-MMP at t = 0. As shown in Figure 1C, M14a decreases quickly followed by a steady state activity of MT1-MMP at a TIMP-2 concentration of 100 nM. The steady state level (M14a_steady_) continues for at least 50,000 sec (Figure S3). The level of M14a_steady_ depends on the TIMP-2 concentration (Figure 1C inset and Figure 1D). At a lower concentration of TIMP-2, M14a_steady_ gradually decreases as the TIMP-2 increases, which is followed by an abrupt decrease around 80 to 120 nM of TIMP-2, and then by an additional gradual decrease after a further increase in TIMP-2. *t_transient_*, which is the half-width of M14a transient activity, has a peak value of 4.2 sec at TIMP-2 of 100 nM, and at lower and higher TIMP-2 concentrations *t_transient_* decreases (Figure S4). These characteristics are almost identical in the absence of MMP-2 (Figure S5).

### Delayed inactivation produces the basis for the sharp transient peak

**Figure 2:**
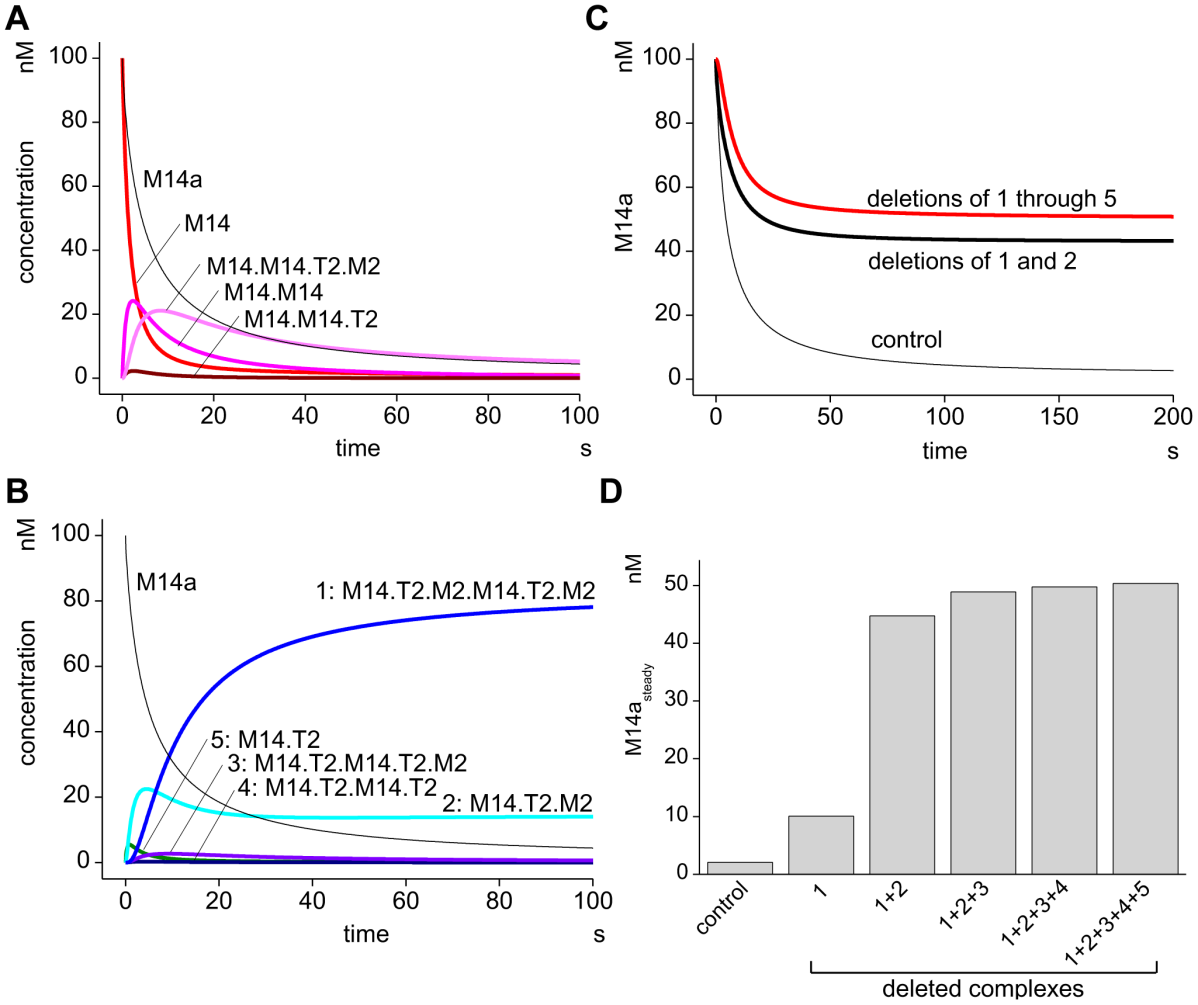
Rapid formation of inactive complexes creates a sharp transient activity of MT1-MMP. (A)Time courses of all complexes comprising M14a, M14, M14.M14, M14.M14.T2, and M14.M14.T2.M2, are shown. The concentration of M14 decreased rapidly in monotonic fashion, concentrations of M14.M14 and M14.M14.T2.M2 resemble biphasic time courses. The concentration of M14.M14.T2 also shows a biphasic time course but is present in negligibly small concentration. (B) Time courses of all inactive complexes of MT1-MMP are shown. Concentrations of M14.T2, M14.T2.M2, and M14.T2.M14.T2.M2 resemble biphasic time courses, while the concentration of M14.T2.M2.M14.T2.M2 increases monotonically until reaching a steady plateau level. The concentration of M14.T2.M2 is also present at a significant level at steady state. The numbers at the left of each complex indicate the complex number. (C) The change in the time course by path-deletion. If pathways leading to the complexes of M14.T2.M2.M14.T2.M2 (deletion 1) and M14.T2.M2 (deletion 2) are deleted, M14a_steady_ is considerably higher than the concentration in the presence of both pathways (control). If we delete all pathways to all of the inactive complexes (deletions 1 through 5), there is an additional increase in M14a_steady_. (D) M14a_steady_ progressively increases together with the increase in the number of deleted complexes.

Next we sought the reason for this sharp transient peak and analyzed the time courses for the concentration changes of active complexes (Figure 2A). M14 quickly decreases within 10 sec after its surface expression, and M14.M14 and M14.M14.T2.M2 have much reduced delayed peaks. In contrast, inactive complexes begin to grow during or after the sharp transient peak of M14a (Figure 2B). Among them M14.T2.M2 resembles the transient peak and it reaches a plateau, and then M14.T2.M2.M14.T2.M2, which is the highest order inactive complex in our model, grows monotonically and reaches a plateau. These computational analyses strongly suggest that the delayed inactivation of active MT1-MMP is the reason for the sharp transient peak of M14a.

To confirm this, we ran simulations that deleted paths to and from inactive complexes and aimed at eliminating transient peaks and increasing the M14a_steady_ level (Figure 2C). If we delete M14.T2.M2.M14.T2.M2 (deletion 1) and M14.T2.M2 (deletion 2), the M14a transient peak is reduced and M14a_steady_ is increased (thick black line) in comparison with the control (thin black line), and if we delete M14.T2.M14.T2.M2 (deletion 3), M14.T2.M14.T2 (deletion4), and M14.T2 (deletion 5) additionally, M14a transient and M14a_steady_ are further decreased and increased, respectively (thick red line). Even after the deletion of all inactive complexes, there still remains a transient in M14a (thick red line). This is because M14a includes a half-inactive complex of M14.M14.T2. The increase in M14a_steady_ by the deletion of inactive complexes (deletions 1 through 5) is clearly seen in Figure 2D, where the M14a_steady_ is progressively increased by the increase in the number of deleted paths. These results clearly indicate that the M14a transient peak seen in our model is due to the delayed inactivation of active MT1-MMP complexes.

### Sharp transient peak is also observed in the presence of the continuous turnover of MT1-MMP, but depends on its rate

In the previous report, we found that there were two pools X and D of MT1-MMP in the invadopodial membrane with turnover time constants of 259 sec and 26.0 sec, respectively [23]. MT1-MMP is docked and internalized to and from these sites, and the turnover of MT1-MMP at these sites was found to be crucial for the ECM degradation. Therefore, we next investigated whether the sharp transient peak of M14a is also observed in the presence of the turnover of MT1-MMP using the same model as that used in the previous report (Figure S6) [23]. ECM degradation was not included in this simulation to compare the transient activity of M14a with those observed in the absence of the turnover of MT1-MMP in the previous section.

**Figure 3:**
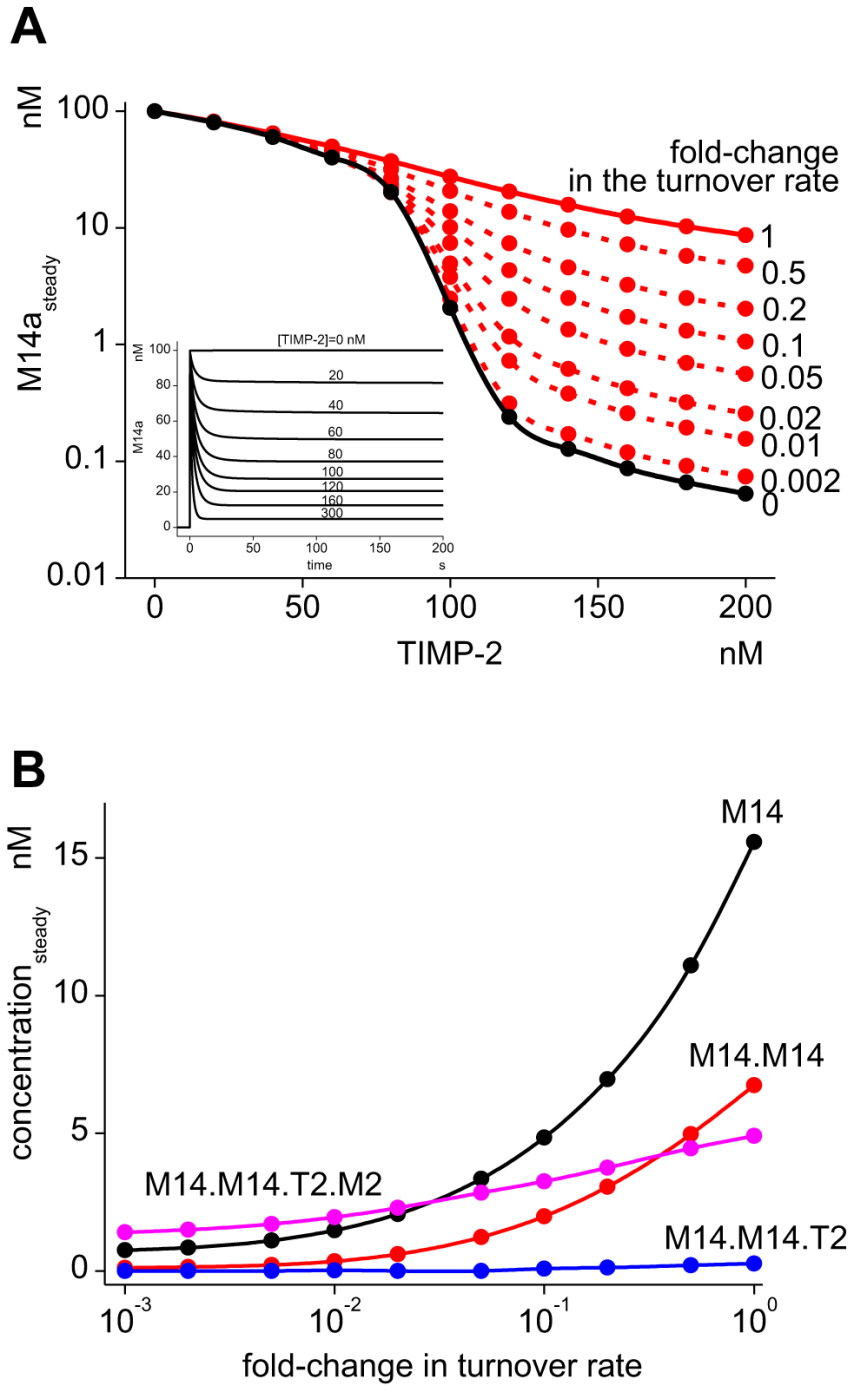
Changes in the M14a_steady_ according to the change in the turnover rate. MT1-MMP undergoes turnover based on our experimental observation [23], and the effect of the change in the turnover rate on M14a_steady_ is investigated by simulations. (A) In the presence of the turnover, M14a_steady_ is considerably higher at TIMP-2 concentrations higher than 80 nM (red continuous line) in comparison to the level in the absence of turnover (black line). If we reduce the turnover rate progressively, M14a_steady_ reaches the level seen in the absence of turnover (red broken lines). (B) Active complexes increase together with the increase in the turnover rate. Among them, M14 displays the most significant increase, followed by M14.M14.

Simulations show the existence of the sharp transient peak also in the presence of the MT1-MMP turnover (inset of Figure 3A). However, the reduction in M14a_steady_ by the increase in TIMP-2 concentrations is greatly reduced (continuous red line in Figure 3A) in comparison with the case in the absence of the turnover (continuous black line). This suggests that M14a_steady_ is modulated by the turnover of MT1-MMP. In fact, M14a_steady_ depends on the turnover rate, as indicated by the broken red lines in Figure 3A, where M14a_steady_ is decreased by the reduction of the turnover rate. It should be noted that the effect of the turnover rate is much more prominent in the region of TIMP-2 concentrations higher than 80 nM. At TIMP-2 concentrations lower than 80 nM, the difference in M14a_steady_ in the presence or absence of turnover is only negligible.

To clarify the mechanism responsible for the different behavior of the M14a transient peak in the presence or absence of MT1-MMP turnover, we next sought to deduce which active complexes are responsible for the increased M14a_steady_ in the presence of the turnover. Although virtually no alteration is seen in M14.M14.T2, there are increases in M14, M14.M14, and M14.M14.T2.M2 at steady state as the turnover rate increases (Figure 3B). Among them, M14 and M14.M14 showed the two largest increases by the increase in the turnover rate. Thus, the increase in monomeric and dimeric MT1-MMP is the reason for the higher steady state level of M14a at a higher turnover rate of MT1-MMP. Thus, the turnover of MT1-MMP abrogates the effective inhibition by TIMP-2 to some extent.

### Sharp transient activity of MT1-MMP is crucial for the ECM degradation

While the ECM-degrading activity of MT1-MMP is high but transient (*t_transient_*<5 sec) in the very beginning after the insertion of MT1-MMP, it is low but long-lasting in the following steady state. Which will contribute the most to ECM degradation, the sharp transient activity or the long-lasting steady state? To explore this, we ran simulations of ECM degradation in the presence or absence of the sharp transient activity. For this purpose, we added a model for the ECM degradation to the one discussed in the previous section, which is the same as before (Figure S14 in [23]). In this model, both MT1-MMP in pools X and D and active MMP-2 (M2_act_) degrade ECM. To realize simulations in which the sharp transient activity was eliminated, we modified the model (See Materials and Methods). Briefly, the MT1-MMP that is inserted into the plasma membrane is apportioned to its complexes according to their relative fractions at steady state. We confirmed that there is no transient peak in M14a or in each of the complexes for pools X and D (Figure S7).

**Figure 4:**
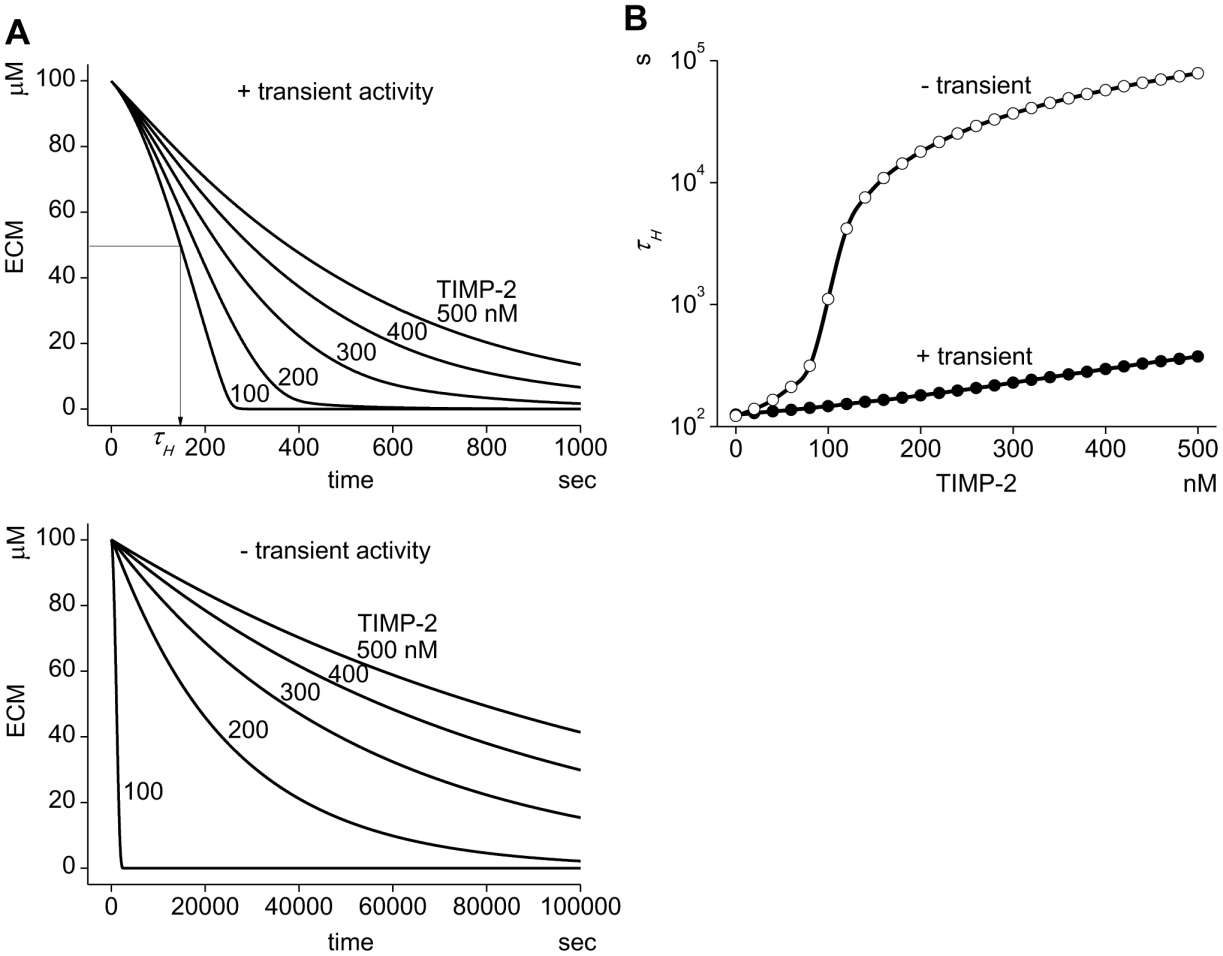
The peak transient activity of MT1-MMP is critical to the effective ECM degradation. (A) In the presence of the sharp transient activity in M14a, ECM is degraded effectively depending on the TIMP-2 concentration. τ*_H_* is defined as the time at which half of ECM is degraded (top panel). If the transient activity is eliminated computationally, the ECM degradation is greatly delayed (bottom panel). (B) The reduction in the efficacy of ECM degradation by the elimination of the sharp transient peak is clearly shown. At TIMP-2 concentrations lower than 40 nM, only a small change in *τ_H_* is seen. At 80 nM of TIMP-2, however, *τ_H_* increases dramatically in the absence of the sharp transient activity. The difference in *τ_H_* is more than two orders of magnitude at TIMP-2 of 500 nM.

The simulated ECM degradation proceeds much faster in the presence of the transient activity than in its absence at all TIMP-2 concentrations from 100 nM to 500 nM (Figure 4A). Thus, the sharp transient activity of MT1-MMP is crucial for the efficient ECM degradation. This situation is clearly shown in Figure 4B, where the time to half-degradation of ECM τ*_H_* is shown at TIMP-2 concentrations from 0 to 500 nM in the presence (closed circles) or absence (open circles) of the transient activity. At all TIMP-2 concentrations except at 0 nM, τ*_H_* is larger in the absence of the transient activity than in its presence. Especially at TIMP-2 concentrations higher than 60 nM, τ*_H_* in the absence of the transient activity shows a dramatic increase, and at TIMP-2 of 500 nM, the difference in τ*_H_* is more than two-orders of magnitude. Thus, τ*_H_* in the absence of the transient activity is increased as the TIMP-2 concentration is increased.

### Pulsatile turnover model resembles the same nature as in the continuous turnover model

In the model discussed above, the turnover of MT1-MMP is simulated as a continuous event. However, MT1-MMP is inserted by vesicular trafficking [5], [21], [23], [26], [29]. This indicates that the actual turnover of MT1-MMP at the invadopodial membrane will proceed in a pulsatile manner. Therefore, we constructed a model for the pulsatile turnover of MT1-MMP. Briefly, the pulsatile insertion intervals for pools X and D are set to be 25.9 and 2.6 sec, respectively, depending on the two time constants observed in our FRAP experiments [23]. These intervals were calculated by assuming that the concentrations of single insertions are 10% of the maximum available docking concentrations for pools X and D, which are 3 nM and 7 nM, respectively (See Materials and Methods for detail).

**Figure 5:**
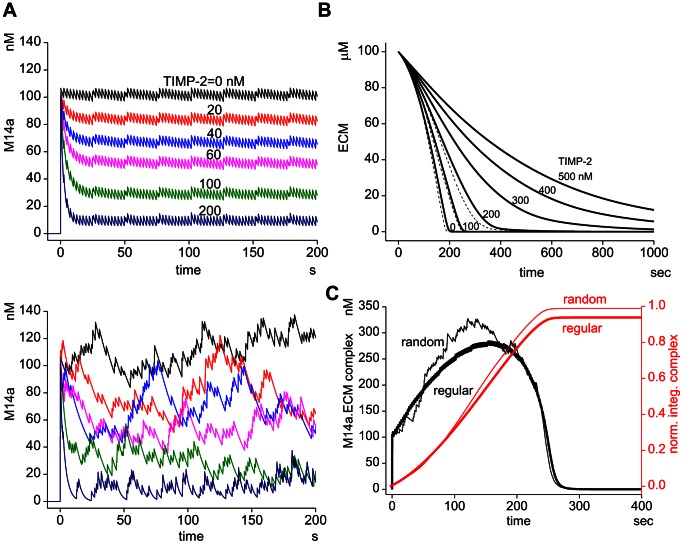
Pulsatile insertion of MT1-MMP. (A) Time courses of M14a in the pulsatile insertion model of MT1-MMP. There are two different intervals in pulsatile insertion of MT1-MMP, which correspond to pools X and D. This results in the double zigzag lines with short (2.6 sec for pool D) and long (25.9 sec for pool X) intervals (top panel). If the intervals of the insertion of MT1-MMP in pools X and D are random instead of regular, M14a follows a considerably random time course (bottom panel). (B) Comparison of time courses for ECM degradation between regular (continuous lines) and random (broken lines) pulsatile insertion intervals. The differences in the time courses are small. (C) If we plot the time courses of complexes of active MT1-MMP and ECM (M14a.ECM) at TIMP-2 of 100 nM, its randomness in the random pulsatile insertion interval is much reduced in comparison to the time course of M14a (bottom panel in A). If the integrated values of M14a.ECM for regular and random pulsatile insertion, which is the direct measure of degraded ECM, are plotted, the curves resemble smooth lines for random pulsatile insertion (thin red line) and regular pulsatile insertion (thick red lines). There is only a small difference between the two.

The time courses of M14a in the pulsatile insertion with regular intervals for TIMP-2 concentrations from 0 to 200 nM are shown in the top panel of Figure 5A. The model schemes are the same as those in Figure S6 except the insertion is pulsatile instead of continuous. The zigzag lines caused by the pulsatile insertion have long and short regular time intervals, which correspond to insertion intervals for pools X (25.9 sec) and D (2.6 sec), respectively. The basic behavior of the M14a time course is the same as that of the continuous insertion model, that is, M14a decreases after the first insertion of MT1-MMP at t = 0, and this decrease becomes larger as TIMP-2 increases. The decreased levels of M14a are identical to those of the continuous insertion model (Figure S8).

It is not known whether the pulsatile MT1-MMP insertion occurs at regular or random intervals. Therefore, we ran simulations of random insertion intervals with their averages corresponding to those of regular insertion, which are 25.9 and 2.6 sec for pools X and D, respectively. The time courses of M14a in random insertion intervals (bottom panel of Figure 5A) differ largely from that of the regular insertion (upper panel).

The simulation results shown above suggest that the regular and random insertion intervals could result in different time courses for ECM degradation. To see this possibility, we ran simulations of ECM degradation with regular and random insertion intervals. Unexpectedly, however, the time course for regular and random insertion protocols did not differ greatly (continuous and broken lines in Figure 5B for regular and random insertion intervals, respectively). These situations are clearly shown in Figure S9, where histograms of τ*_H_* are shown at TIMP-2 concentrations of 0, 200, and 400 nM with 120±9.66, 173±16.2, 277±32.9 sec. The SDs are around 10% of the average. The same situation is also true when amount instead of interval of MT1-MMP at single pulsatile insertion is varied randomly (Figure S10).

The simulation results discussed above clearly show that the time course of ECM degradation does not change considerably despite the large variability in the time course of M14a. To explore the reason, we investigated the time course of the M14a.ECM complex ( = M14.ECM+M14.M14.ECM+M14.M14.T2.ECM+M14.M14.T2.M2.ECM), because the concentration of this complex determines the rate of ECM degradation. The random insertion protocol gives higher or lower concentrations of M14a.ECM (black thin line in Figure 5C) in comparison to the regular insertion protocols (black thick line). If we take the integrated concentration of the complex, which is directly related to the amount of degraded ECM and hence the time course of ECM degradation, the difference in the amount of degraded ECM between regular and random interval insertion is reduced (red lines in Figure 5C). Taken together, the difference in the time course of ECM degradation and hence τ*_H_* is much reduced even if the insertion intervals or the amount at a single insertion of MT1-MMP are randomly changing.

### Large concentrations and longer intervals between insertions provide most effective ECM degradation

**Figure 6:**
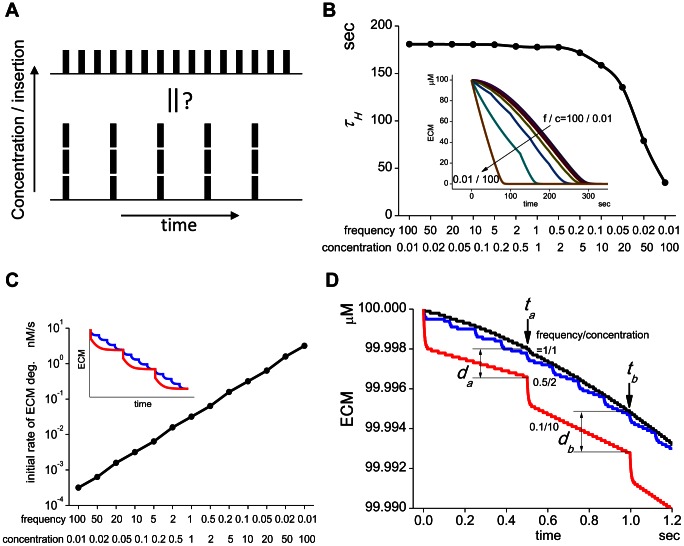
The effect of MT1-MMP concentration at each insertion while keeping time-averaged insertion amount unchanged. (A)Two examples of pulsatile insertion of MT1-MMP. The upper example shows small but frequent insertions, while the lower example shows three-times larger insertions but with one-third of the insertion frequency. In both cases, the time-averaged insertion amount of MT1-MMP is unchanged. The problem is whether these two regimens result in the same time courses of ECM degradation. (B) There is no change in *τ_H_* by the fold-increase in frequency/concentration of pulsatile insertion from 100/0.01 to 0.2/5, while it decreases with the change from 0.1/10 to 0.01/100. This indicates that ECM degradation proceeds faster in cases of higher MT1-MMP concentration in a single vesicle with longer insertion intervals, even if the time-averaged amount of MT1-MMP is not changed. The inset shows the time courses of ECM degradation. At the control condition (1/1), the frequency/concentration are (1/25.9 sec)/3 nM and (1/2.6 sec)/7 nM for pools X and D, respectively. (C) There is a positive correlation between the initial rate of ECM degradation and the fold-change in insertion frequency/concentration. It is expected that the ECM remaining at the next insertion is the same even if the frequency/concentration regimen is different (inset). (D) At a higher frequency/smaller concentration regimen, the ECM concentration remaining at the next insertion is almost unchanged with the change in the regimen (black and blue lines). However, at lower frequency/higher concentration regimens, there are significant differences in the remaining ECM at the time of later insertions (*t_a_* and *t_b_*). This results in the larger value of (inserted MT1-MMP)/(remaining ECM), and a larger amount of ECM is degraded with the same amount of inserted MT1-MMP at later insertions. Thus, the difference in the remaining ECM (*d_a_* and *d_b_*) increases at later insertions.

In the previous section we assumed the concentrations of single pulsatile insertions were 10% of the maximum available docking concentrations for pools X and D. However, the amount of MT1-MMP in a single vesicle is not known. Therefore, it is important to investigate the effect of different concentrations in single insertions. To this purpose, we set the time-averaged amount of the insertions unchanged but used different frequencies and amounts in a single insertion. The question is whether the two regimens shown in Figure 6A result in the same ECM degradation efficacy.

To test this question, we ran simulations by changing the frequency and concentrations while keeping the average amount unchanged. In the control condition, the frequency/concentration for pools X and D are (1/25.9 /sec)/3 nM and (1/2.6 /sec)/7 nM, respectively. In the range from frequency/concentration of 100/0.01-folds of the control (1/1) to 0.2/5-folds, there is virtually no change in the τ*_H_* (Figure 6B). This situation is also shown in the time course of ECM degradation, where all 13 sets of frequency/concentration are shown, and the time courses for 9 sets from 100/0.01 to 0.2/5 are overlapping (Figure 6B inset). When the concentration is larger than fivefold of the control, however, τ*_H_* begins to decrease despite the average insertion amount is the same. Thus, the ECM-degrading efficacy is higher for lower frequency/higher concentration regimens.

These results seem strange, since the average amount of insertion was not changed. To explore the reason, we analyzed the change in the initial rate of ECM degradation, which is the rate of the ECM degradation at t = 0, by the change in frequency/concentration (Figure 6C). The initial rate increases by the increase in the concentration as it was expected to do. Then, it would be expected that the initial higher rate of ECM degradation at the lower frequency/higher concentration regimen would be gradually reduced, and that the ECM concentration at the next insertion in this regimen would coincide with that of the higher frequency/lower concentration regimen (blue and red lines in the inset of Figure 6C).

To explore whether or not this is the case, we analyzed the time courses of ECM degradation at a very early stage (Figure 6D). The expectation was almost met for the higher frequency/lower concentration regimen (black and blue lines for frequency/concentration of 1/1 and 0.5/2, respectively). If the frequency/concentration is changed to 0.1/10 (red line), however, the ECM concentration at the second insertion time (*t_a_*) is significantly lower (the difference is *d_a_*). Thus, the expectation shown in the inset of Figure 6C is not met for the lower frequency/higher concentration regimen. If we look at Figure 6D carefully, the difference in the ECM concentration is larger at later times of insertion (*d_a_*<*d_b_* at time *t_b_*), and the slope of the curve is larger during *t_a_* to *t_b_* than from 0 to *t_a_*. This indicates that the efficacy of the same concentration of inserted MT1-MMP is larger at later times of insertion. Since the ECM concentration at later times is decreased by the degradation, the relative value of inserted MT1-MMP/ECM and hence the efficacy to degrade ECM will be higher at later times. This will cause more efficient ECM degradation for lower frequency/higher concentration regimens, and result in a smaller τ*_H_*. Thus, the equality shown in Figure 6A does not hold at later times, and the difference in the ECM degradation efficacy is significantly higher at lower frequency/higher concentration regimens, even if the average insertion amount is the same.

### Significance of sharp transient activity of MT1-MMP for ECM degradation is also true for ECM degradation in spatially distributed environment

**Figure 7:**
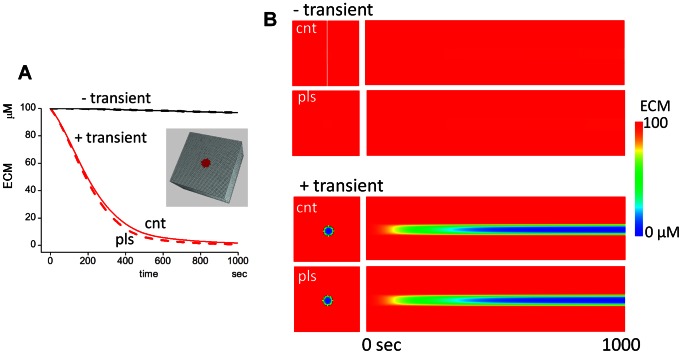
Spatio-temporal simulation of ECM degradation in the presence and absence of the sharp transient activity. (A) ECM degradation in the 3D model. The 3D space was cuboid with axes of 5 µm×5 µm and 3 µm (inset). The space was divided into 51×51×1 compartments. One surface of the space was assumed to be the ventral surface of a cancer cell, and ECM was present all along the space. We set 49 compartments at the center as an invadopodium (shown in red in the inset) whose diameter was about 0.9 µm. The time courses for continuous (continuous lines) and regular pulsatile (broken lines) insertion in the presence (red lines) and absence (black lines) of the sharp transient activity at TIMP-2 of 200 nM are shown. In both cases, the sharp transient activity in M14a plays a critical role, and virtually no ECM degradation is seen in its absence. (B) Line-scans showing spatio-temporal change in the ECM degradations. Spatial profile was taken along a white line. There is virtually no difference between continuous and pulsatile insertion in the spatio-temporal dynamics of ECM degradation, but there is considerable difference between in the present and absence of transient M14a activity. No appreciable ECM degradation is seen when transient activity of M14a is eliminated.

All of the simulations discussed above were performed without any spatial distribution. Next we investigated the significance of the sharp transient activity both for continuous and pulsatile insertions in a spatially distributed model. In the pulsatile insertion, both the interval and the concentration of insertions were regular. The spatially distributed model of ECM degradation was the same as that discussed earlier [23]. Briefly, the extracellular space of 5×5×3 µm, which is assumed to be filled with ECM proteins, is divided into 51×51×1 compartments of identical size. At the center of one surface of the extracellular space, the focal degradation of ECM was defined (red compartments in the inset of Figure 7A). Models for the activation of MT1-MMP, its turnover, the activation of proMMP2, and ECM degradation by MT1-MMP were embedded to these compartments. ECM degradation by MMP-2 and deactivation of MMP-2 by TIMP-2 were embedded to all compartments. proMMP-2, MMP-2, TIMP-2 and TIMP-2.MMP-2 complex are diffusing species.

Black lines in Figure 7A show the time course of ECM degradation in the absence of the sharp transient activity. Virtually no ECM degradation is seen neither in continuous (continuous line) nor pulsatile insertions (broken line) at TIMP-2 of 200 nM. In the presence of the sharp transient activity (red lines), however, ECM degradation proceeds both in continuous and pulsatile insertions with almost identical time courses. If we plot the spatio-temporal profile of the ECM degradation along a vertical white line crossing the invadopodial region shown in Figure 7B, virtually no ECM degradation is seen in the absence of sharp transient activity both in continuous and pulsatile insertions. In the presence of the sharp transient activity, however, ECM degradation proceeds significantly in both insertion regimens. The profiles of ECM degradation are almost identical in continuous and pulsatile insertions. These simulation results clearly indicate a crucial role for the transient peak in ECM degradation also in a spatially distributed environment.

## Discussion

The dynamics of the activity of MT1-MMP at the surface of invadopodia is currently difficult to measure experimentally. In this study we have analyzed the activity of MT1-MMP in terms of ECM degradation. First, our simulation suggests the existence of a sharp transient peak with a half-width of less than 5 sec followed by a steady state level of active MT1-MMP complexes. Second the steady state level of MT1-MMP complexes depends on the TIMP-2 concentration, being lower at higher TIMP-2 concentrations. Third, we found that this sharp transient peak is critical for the effective ECM degradation. If this sharp transient peak was eliminated computationally, the efficacy of ECM degradation was dramatically reduced. Fourth, in the pulsatile insertion of MT1-MMP, the effect of random insertion intervals or random insertion concentrations of MT1-MMP is small, resulting in a small variance in τ*_H_* in comparison to the regular insertion regimen. Finally, the insertion of MT1-MMP at lower frequency/higher concentration resulted in a faster ECM degradation even if the time-averaged amount of insertion is the same. Thus we have shown a number of critical characteristics of MT1-MMP dynamics in the ECM degradation.

**Figure 8:**
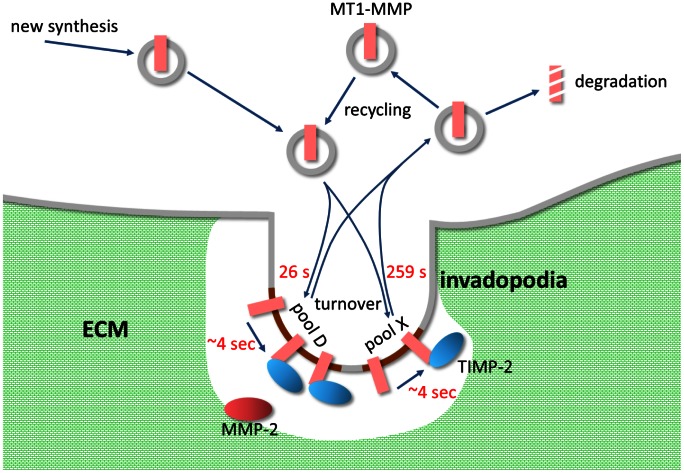
The dynamics of MT1-MMP for degrading ECM at invadopodia. There are two docking sites in the invadopodial membrane to which MT1-MMPs are inserted (pools D and X). The turnover rates of MT1-MMP in pools D and X are 26.0 sec and 259 sec, respectively, and MT1-MMP to these pools is transported to these pools by vesicular trafficking. MT1-MMP inserted into these pools is subjected to ECM degradation and also to inhibition by the endogenous inhibitor TIMP-2. The present study has shown a quick inhibition by TIMP-2, and hence the half-life for newly inserted MT1-MMP to maintain ECM-degrading activity is only about 4 sec depending on the TIMP-2 concentration. This short life-time is compensated by the quick turnover of MT1-MMP resulting in the effective degradation of ECM at invadopodia.

These results are summarized in Figure 8. There are two docking sites in the membrane of invadopodia into which MT1-MMPs are inserted. We call these sites pools D and X. The turnover rates of MT1-MMP in pools D and X are 26.0 sec and 259 sec, respectively [23]. MT1-MMP is transported to these pools by vesicular trafficking via various routes in the cytoplasm. MT1-MMP inserted into these pools is subjected to ECM degradation and also to inhibition by the endogenous inhibitor TIMP-2. This inhibition is fast, and hence the half-life for newly inserted MT1-MMP to possess ECM-degrading activity is only about 4 sec depending on the TIMP-2 concentration (Figures 1, 2 and S4). This short half-life is compensated by the fast turnover of MT1-MMP leading to the effective degradation of ECM at invadopodia. It should be noted that the model is not a simple enzymatic reaction system, in which one enzyme catalyzes a reaction of one substrate. MT1-MMP catalyzes both of the processing of proMMP-2 and at the same time the degradation of ECM. Thus the present model describes a one enzyme-two substrate system. This will result in the rivalry between two enzymatic reactions.

In the previous report, we showed that reduction of the turnover rate of MT1-MMP is one possible therapeutic target [23]. This has been confirmed in the present study, since the steady-state concentration of M14a at higher TIMP-2 concentrations is dramatically decreased by a reduction of the turnover rate (Figure 3A). In addition, the effectiveness of a combined treatment with an increased TIMP-2 concentration and elimination of the sharp transient peak is shown in Figure 4B. The difference in τ*_H_* between the presence and absence of the sharp transient activity is much larger at higher TIMP-2 concentrations. Thus, at a TIMP-2 concentration of 500 nM, the difference in τ*_H_* is more than two orders of magnitude. It is thought that the increased TIMP-2 concentration caused by a drug results in a reduced ECM degrading activity. However, it is not the case in the presence of the sharp transient activity as shown in Figure 4B, because the sharp transient activity as short as 4 sec is effective to degrade ECM. The prevention of this sharp transient activity will require a drug with much higher affinity and much faster binding kinetics to MT1-MMP than TIMP-2.

On the other hand, an abrupt increase in the inserted MT1-MMP concentration has only a small effect in ECM degradation. This is shown by histograms of τ*_H_* for random insertion intervals and concentrations with relatively small variance (Figures S9 and S10). The vesicular content of MT1-MMP and its insertion intervals can be randomly fluctuated, and insertions at unexpectedly large or unexpectedly short intervals will then occur. From our simulation results, however, its effect is smaller than expected. In fact, an abrupt large amount of insertion has only a small effect on the time course of ECM degradation (Figure S11).

In contrast, the concentration of MT1-MMP in progressively inserted vesicles has a significant effect on ECM degradation. Although the same ECM degradation efficacy was expected for different sets of insertion frequency/concentration with identical time-averaged amount of inserted MT1-MMP, our simulation results have shown that this is not the case, and instead ECM degradation is much efficient at higher concentration and lower frequency regimens (Figure 6B). The time of half-degraded ECM τ*_H_* was shorter with the higher vesicular content, because of the increased ratio of inserted MT1-MMP/residual ECM. This suggests the importance of reducing vesicular content of MT1-MMP in order to prevent accelerated ECM degradation.

If we look at the spatio-temporal dynamics of free TIMP-2 concentration, we found its large spatial gradient at an initial TIMP-2 concentration of 20 nM with increasing spatial gradient with time (Figure S12A). This indicates that the sharp transient peak is a phenomenon that is also observed in the limited accessibility of TIMP-2 at invadopodia. To further confirm this, we ran simulations with different diffusion coefficients from 2×10^−11^ to 2×10^−19^ m^2^/s and with an increased number of invadopodia (Figures S12B and C). The sharp transient peak was still observed at conditions with different diffusion coefficients both in single- and five-compartment cases. It was suggested that the TIMP-2 concentration is lower at invadopodia around the cell center than at lamellipodia around the cell periphery, because of the limited accessibility of TIMP-2 at the cell center [30]. Our simulation results indicate that the sharp transient peak exists even at the situation of limited accessibility of TIMP-2 at the invadopodia.

The surface MT1-MMP is subjected to the ectodomain shedding. Fragments of MT1-MMP diffuse freely and bound with TIMP-2. Thus it acts as a scavenger of TIMP-2 and this reaction can be an additional mechanism limiting the availability of TIMP-2 at invadopodia. However, there is only a negligible effect both on the sharp transient peak and the ECM-degrading activity in our simulation (Figure S13). Thus in this report, we further characterized the model that we reported on earlier [23], and found important characteristics in the activity of MT1-MMP in regard to ECM degradation that suggest potential therapeutic targets.

## Materials and Methods

### Experiment

The SCC61 cell lines, which have been described previously [31], were maintained in DMEM with 20% fetal bovine serum with 0.4 mg/ml hydrocortisone. The matrix degradation assay was performed as before [23]. Briefly, fibronectin (BD Biosciences) was labeled with Dylight 633 (Fisher) by dialysis in borate buffer [0.17 mol/L borate, 0.075 mol/L NaCl (pH 9.3)], and the buffer was changed to PBS and dialyzed extensively for 3 to 4 days. A solution of 2.5% gelatin/2.5% sucrose in PBS was added to dishes to coat MatTek, followed by crosslinking with 0.5% glutaraldehyde in PBS. A solution of 50 µg/mL solution of fluorescence-labeled fibronectin was incubated with the crosslinked gelatin in MatTek dishes in the dark for 1 h. The dish was sterilized with 70% ethanol, washed with PBS, and equilibrated with medium [DMEM supplemented with 15% FBS and 5% Nu-Serum (BD)] for 30 min before the addition of cells. A total of 7×10^4^ cells were suspended in 2 mL of medium containing 100 µM EGF.

### Construction of computational model

The models were constructed using A-Cell [32], [33], and all models can be downloaded from http://www.ims.u-tokyo.ac.jp/mathcancer/A-Cell/index_e.html. The model for continuous insertion of MT1-MMP is the same as the model described earlier [23]. Briefly, the model has two independent pools of X and D that dock MT1-MMP, where docked MT1-MMP interacts with TIMP-2 and forms the ternary complex (MT1-MMP-TIMP-2-MMP-2). MT1-MMP is internalized and inserted. The model includes the activation of proMMP-2 by MT1-MMP, MMP-2 inactivation by TIMP-2, and ECM degradation by MT1-MMP and MMP-2 at both pools. In the spatio-temporal simulations, biochemical reactions were embedded into the 3D shape. Details of the models and parameter values are shown in Figures S2 and S6 and Tables S1 and S2. In the A-Cell model, MT1-MMP, TIMP-2, and MMP-2 were designated as M14, T2, and M2 for simplicity.

### Computational elimination of the sharp transient

In our model of MT1-MMP dynamics, the ECM-degrading activity resembles a sharp transient activity followed by a steady state level (Figure 1C). In order to elucidate the role of this sharp transient peak in ECM degradation, we computationally eliminate it by distributing amounts of newly inserted MT1-MMP among all complexes according to their fractions at the steady state level. This method for the elimination of the sharp transient peak was applied both for continuous and pulsatile insertion.

### Pulsatile insertion model

Simulations for pulsatile insertion were run both for regular and random intervals. In the regular interval model, the insertion interval for pool X was so calculated such that the time-averaged amount of insertion was the same as that for the continuous insertion model using the following equation:
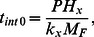
(1)where *PH_x_*, *k_X_* and *M_F_* are, respectively, amount of MT1-MMP at a single insertion, the rate of insertion in the continuous insertion model, and the concentration of free docking sites for MT1-MMP on the invadopodial membrane, respectively. However, no insertion will occur if all docking sites are occupied by MT1-MMP and there is no available site. Therefore, Eq.1 should be modified as follows:
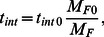
(2)where *M_F0_* is the concentration of maximum docking sites. The same method was applied for pool D, and we got *t_int0_* for pools X and D of 25.9 sec and 2.6 sec, respectively if we assume the concentrations of single insertions of 10% to the maximum available docking concentration for pools X and D, which are 3 nM and 7 nM. For the simulation of regular insertion, MT1-MMP was inserted at a constant interval of the calculated value above. For the simulation of random insertion, MT1-MMP was inserted at random intervals or random concentrations, whose time-average inserted amount of MT1-MMP was the same as in regular insertions. The simulation program was modified for the use of random insertion.

### Simulations

A reaction-diffusion simulation program in C language was automatically generated by A-Cell from the constructed model, and compiled using Intel C++ Studio XE 2011 for Linux. The differential equations were numerically integrated by the fourth-order Runge-Kutta method. Simulations were run on a Linux-based system with Intel Xeon X5680 3.33 GHz.

## Supporting Information

Figure S1 **State transition diagram for the complex formation of MT1-MMP, TIMP-2 and MMP-2.** All possible complexes together with monomers of MT1-MMP (MMP14), TIMP-2, and MMP-2 are shown. A pair of two lines of the same color to a complex indicates transitions from two components of the complex. In case of dimerization, a single line is drawn.
(TIF)

Figure S2 **A-Cell model for the dynamics of MT1-MMP.** The state transition diagram of Figure S1 is modeled using A-Cell software showing its graphical representation of chemical reactions between MT1-MMP (M14), TIMP-2 (T2), and MMP-2 (M2).
(TIF)

Figure S3 **Steady state activity of MT1-MMP.** The steady state activity of MT1-MMP after a transient peak continues for more than 50,000 sec in the absence of ECM.
(TIF)

Figure S4 **The half-width of the transient activity of M14a as a function of TIMP-2 concentration.** The half-width of the transient activity, *t_transient_* is not a monotonic function of TIMP-2 concentration: it has a single peak at TIMP-2 of 100 nM, and it is smaller at lower or higher TIMP-2 concentrations in our simulation conditions, where the initial concentrations were 100 nM for both MT1-MMP and MMP-2.
(TIF)

Figure S5 **Time course of M14a and its TIMP-2-dependency of M14a_steady_ in the absence of MMP-2.** (A) Time courses of M14a are almost identical in the presence or absence of MMP-2 (the inset of Figure 1C). (B) M14a_steady_ is slightly higher in the absence of MMP-2 at higher TIMP-2 concentration. This is because in the absence of MMP-2, the formation of three inhibitory complexes M14.T2.M2, M14.T2.M14.T2.M2 and M14.T2.M2.M14.T2.M2 are blocked. Thus, M14a_steady_ is slightly increased in the absence of MMP-2.
(TIF)

Figure S6 **A-Cell model for the dynamics of MT1-MMP including its turnover for pools X and D.** In this model, the continuous insertion of MT1-MMP is assumed as in the previous report [23].
(TIF)

Figure S7 **Simulated elimination of the sharp transient activity in the model for the ECM degradation.** The model for the ECM degradation is the same as that above except for the elimination of the sharp transient activity, which was realized by distributing newly inserted MT1-MMP and its complexes in proportion to their steady state levels for insertion. (A) There is no transient peak in the time course of M14a at TIMP-2 of 0, 80, 100, 120, 200, and 500 nM. (B) No transient peak is seen in each complex comprising M14a from pools D and X, which are indicated by suffixes D and X, respectively (e.g. M14D and M14X.M14X, etc.). Complexes M14D and M14x.M14x.T2.M2 are overlapping. TIMP-2 is 100 nM.
(TIF)

Figure S8 **Pulsatile insertions and comparison with continuous insertion.** Time courses of M14a are drawn in different colors for TIMP-2 concentration, which are overlapped by continuous insertion model in gray lines.
(TIF)

Figure S9 **Histogram of** ***τ_H_*** **for pulsatile insertion with random intervals and regular content of MT1-MMP.** The average±SD of *τ_H_* for degrading ECM at TIMP-2 of 0, 200, and 400 nM are 120±9.66, 173±16.2, and 277±32.9 sec, respectively. SD by pulsatile insertion is relatively small ranging from 8.05 to 11.9% of the average.
(TIF)

Figure S10 **Pulsatile insertion with random amount of MT1-MMP at a single insertion.** (A) The time courses of M14a for TIMP-2 from 0 to 200 nM resemble the same random behavior as in the insertion at random intervals with regular amount of MT1-MMP. (B)There are some differences in the time course of ECM degradation, but they are not large. (C) The histograms of *τ_H_* show small variances as in Figure S10.
(TIF)

Figure S11 **Abrupt insertion of unexpectedly large content of MT1-MMP.** To see the effect of abrupt insertion event of large MT1-MMP content vesicle, we ran simulations for insertion of large MT1-MMP content at the time of *τ_H_* in the course of ECM degradation as a representative event of abrupt insertion. The MT1-MMP content was 5-, 10-, 20-times of the control.
(TIF)

Figure S12 **Spatio-temporal profile of TIMP-2.** (A) At TIMP-2 concentration of 20 nM, there is a large spatial gradient in TIMP-2, and it grows with time. (B)(C) A sharp transient peak in MT1-MMP activity is observed at diffusion coefficient of TIMP-2 from 2×10^−19^ to 2×10^−11^ m^2^/s both in single invadopodium and five invadopodia simulations. Insets show spatial distribution of TIMP-2 at 200 sec. These simulation results indicate the existence of the sharp transient activity of MT1-MMP even in the limited accessibility of TIMP-2 at invadopodia.
(TIF)

Figure S13 **Effect of ectodomain shedding of MT1-MMP on the ECM degradation.** (A) A model for the ectodomain shedding of MT1-MMP. The rate constant of shedding, *k_shed_*, is the same as used before [34]. (B) The difference in the time course of M14a in the absence and presence of the ectodomain shedding is small. (C) There is only a small difference in the time courses of ECM degradation in the absence and presence of ectodomain shedding.
(TIF)

Table S1 **Parameters for analyzing dynamics of MT1-MMP in the temporal simulations.** Parameter values in this table correspond to the model shown in Figure S2. All parameter values not listed in the table are set at 0 initially.
(PDF)

Table S2 **Parameters for analyzing dynamics of MT1-MMP with pools X and D in the temporal simulations.** Parameter values in this table correspond to the model shown in Figure S6. All parameter values not listed in the table are set at 0 initially.
(PDF)
