# Correction: Critical Role of Transient Activity of MT1-MMP for ECM Degradation in Invadopodia

**DOI:** 10.1371/annotation/b41ed85a-11d1-483a-a7ac-9d06f0c8669a

**Published:** 2013-06-12

**Authors:** Ayako Watanabe, Daisuke Hoshino, Naohiko Koshikawa, Motoharu Seiki, Takashi Suzuki, Kazuhisa Ichikawa

The second author's name was spelled incorrectly. The correct name is: Daisuke Hoshino. The correct citation is: Watanabe A, Hoshino D, Koshikawa N, Seiki M, Suzuki T, et al. (2013) Critical Role of Transient Activity of MT1-MMP for ECM Degradation in Invadopodia. PLoS Comput Biol 9(5): e1003086. doi:10.1371/journal.pcbi.1003086

