# Supplementary figures and images for: Critical Role of Transient Activity of MT1-MMP for ECM Degradation in Invadopodia
Source: PLoS Comput Biol. 2013 May 30;9(5):e1003086. doi: 10.1371/journal.pcbi.1003086 (PMC3667784; doi:10.1371/journal.pcbi.1003086)

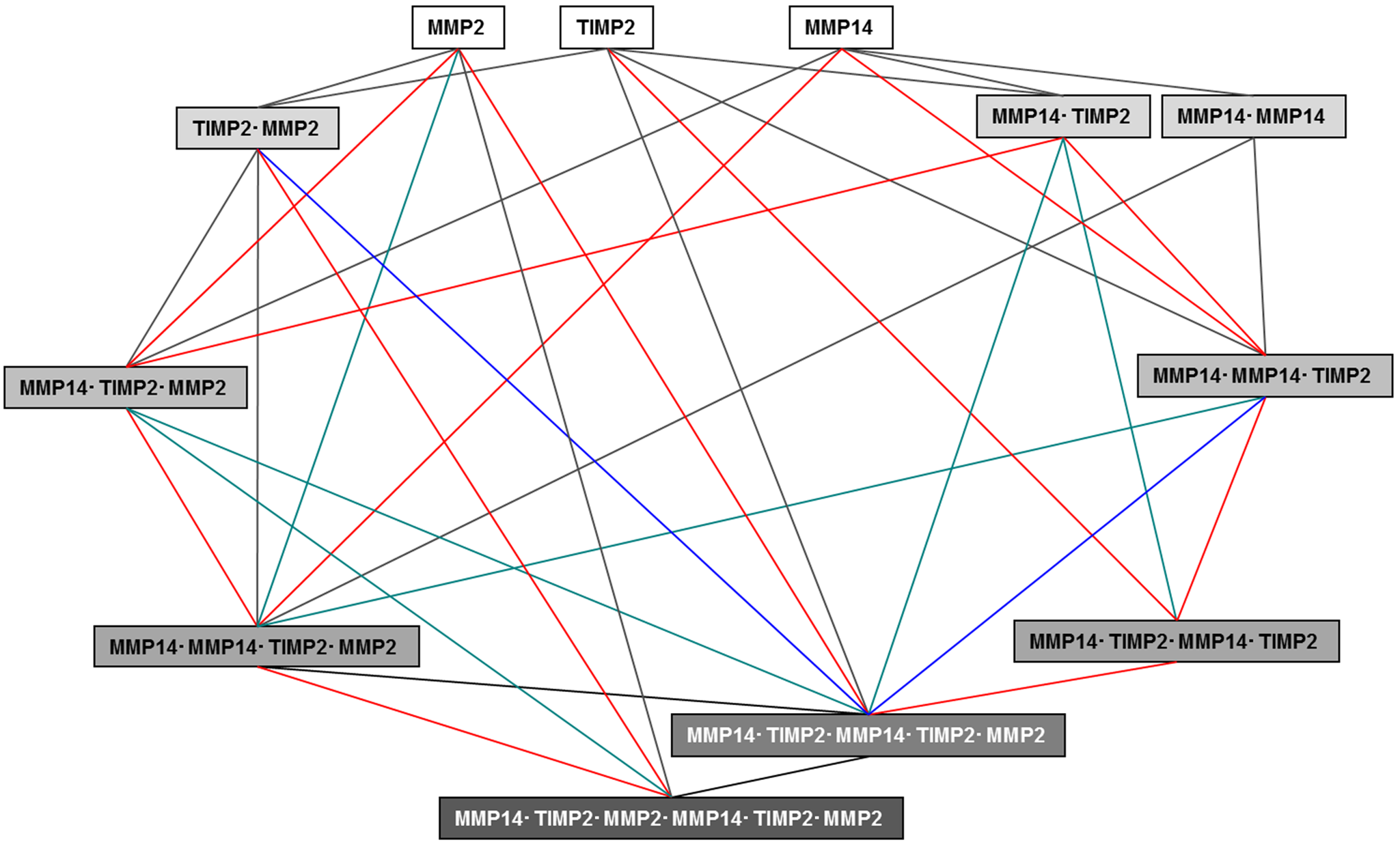

Supplement: Figure S1 — State transition diagram for the complex formation of MT1-MMP, TIMP-2 and MMP-2. All possible complexes together with monomers of MT1-MMP (MMP14), TIMP-2, and MMP-2 are shown. A pair of two lines of the same color to a complex indicates transitions from two components of the complex. In case of dimerization, a single line is drawn. (TIF) [file pcbi.1003086.s001.tif]

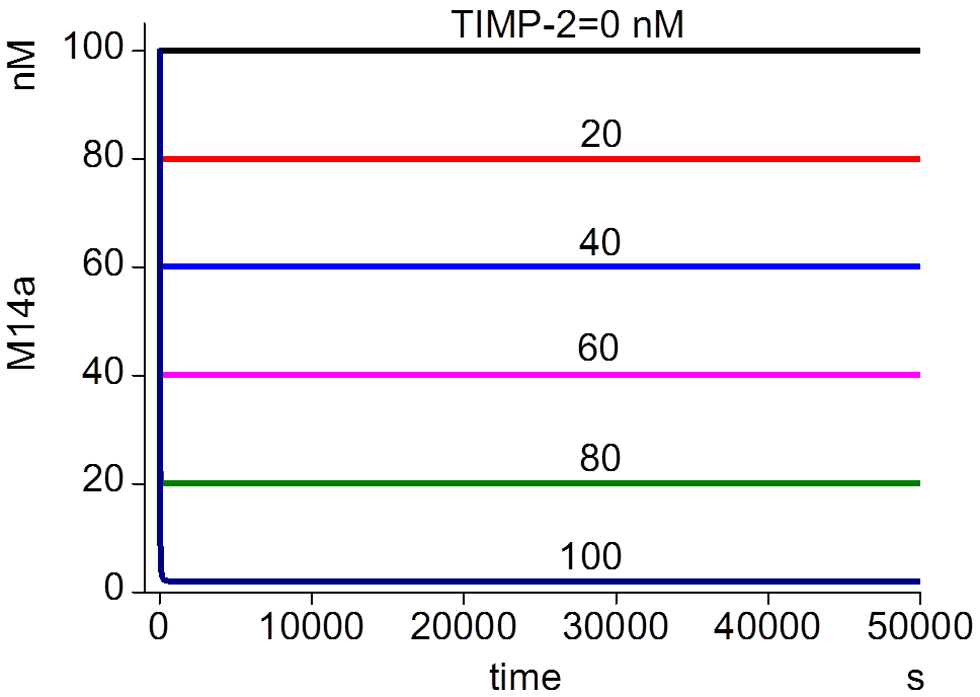

Supplement: Figure S3 — Steady state activity of MT1-MMP. The steady state activity of MT1-MMP after a transient peak continues for more than 50,000 sec in the absence of ECM. (TIF) [file pcbi.1003086.s003.tif]

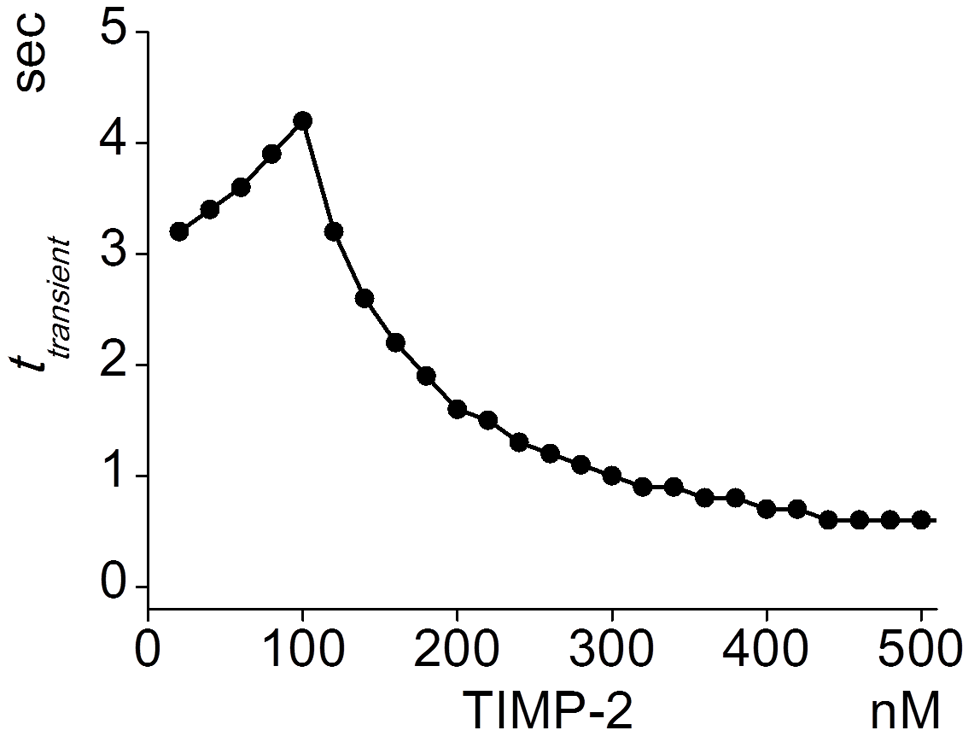

Supplement: Figure S4 — The half-width of the transient activity of M14a as a function of TIMP-2 concentration. The half-width of the transient activity, ttransient is not a monotonic function of TIMP-2 concentration: it has a single peak at TIMP-2 of 100 nM, and it is smaller at lower or higher TIMP-2 concentrations in our simulation conditions, where the initial concentrations were 100 nM for both MT1-MMP and MMP-2. (TIF) [file pcbi.1003086.s004.tif]

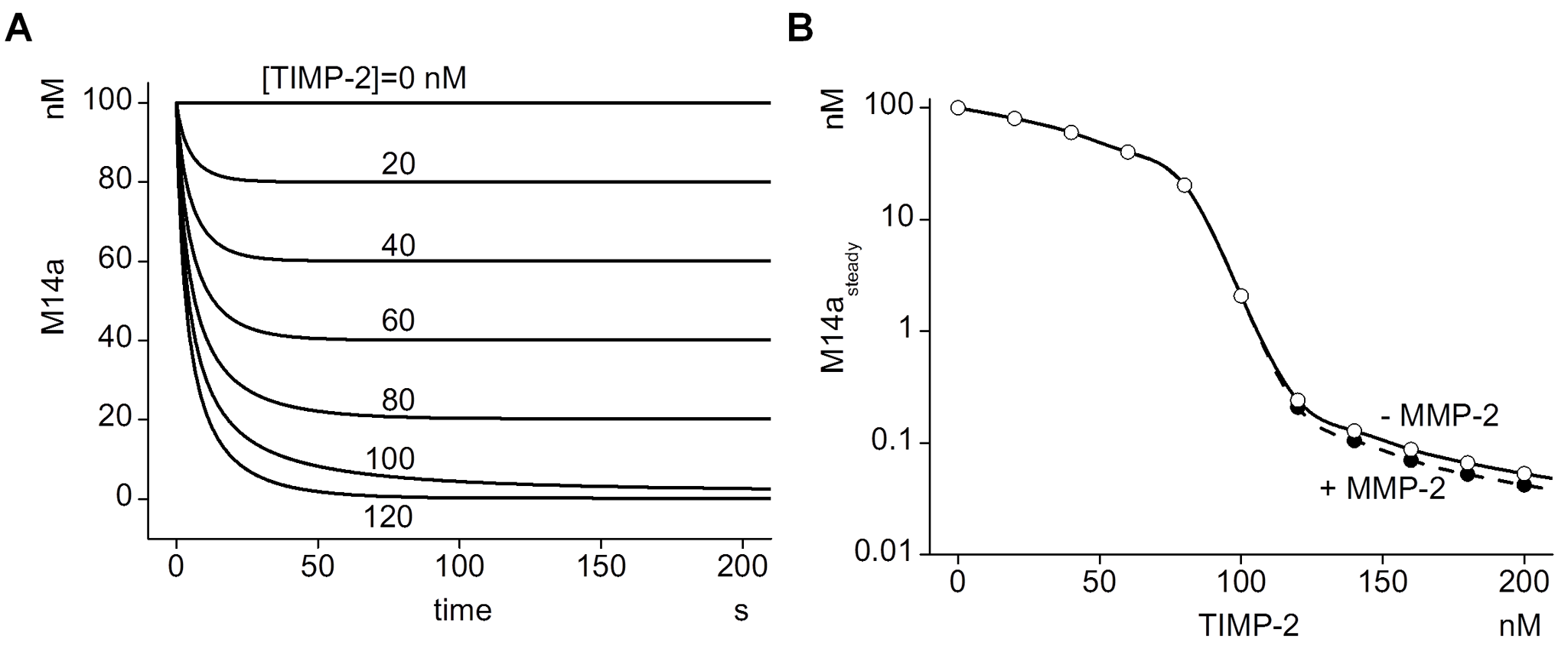

Supplement: Figure S5 — Time course of M14a and its TIMP-2-dependency of M14asteady in the absence of MMP-2. (A) Time courses of M14a are almost identical in the presence or absence of MMP-2 (the inset of Figure 1C). (B) M14asteady is slightly higher in the absence of MMP-2 at higher TIMP-2 concentration. This is because in the absence of MMP-2, the formation of three inhibitory complexes M14.T2.M2, M14.T2.M14.T2.M2 and M14.T2.M2.M14.T2.M2 are blocked. Thus, M14asteady is slightly increased in the absence of MMP-2. (TIF) [file pcbi.1003086.s005.tif]

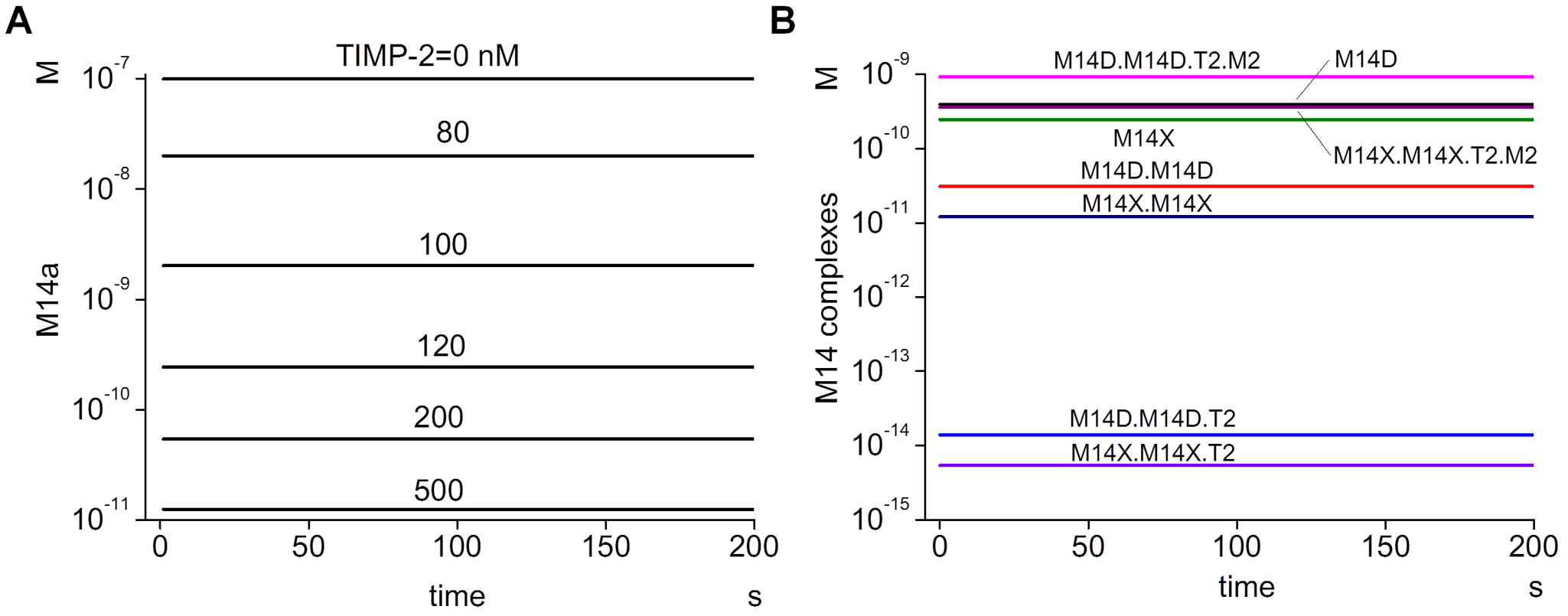

Supplement: Figure S7 — Simulated elimination of the sharp transient activity in the model for the ECM degradation. The model for the ECM degradation is the same as that above except for the elimination of the sharp transient activity, which was realized by distributing newly inserted MT1-MMP and its complexes in proportion to their steady state levels for insertion. (A) There is no transient peak in the time course of M14a at TIMP-2 of 0, 80, 100, 120, 200, and 500 nM. (B) No transient peak is seen in each complex comprising M14a from pools D and X, which are indicated by suffixes D and X, respectively (e.g. M14D and M14X.M14X, etc.). Complexes M14D and M14x.M14x.T2.M2 are overlapping. TIMP-2 is 100 nM. (TIF) [file pcbi.1003086.s007.tif]

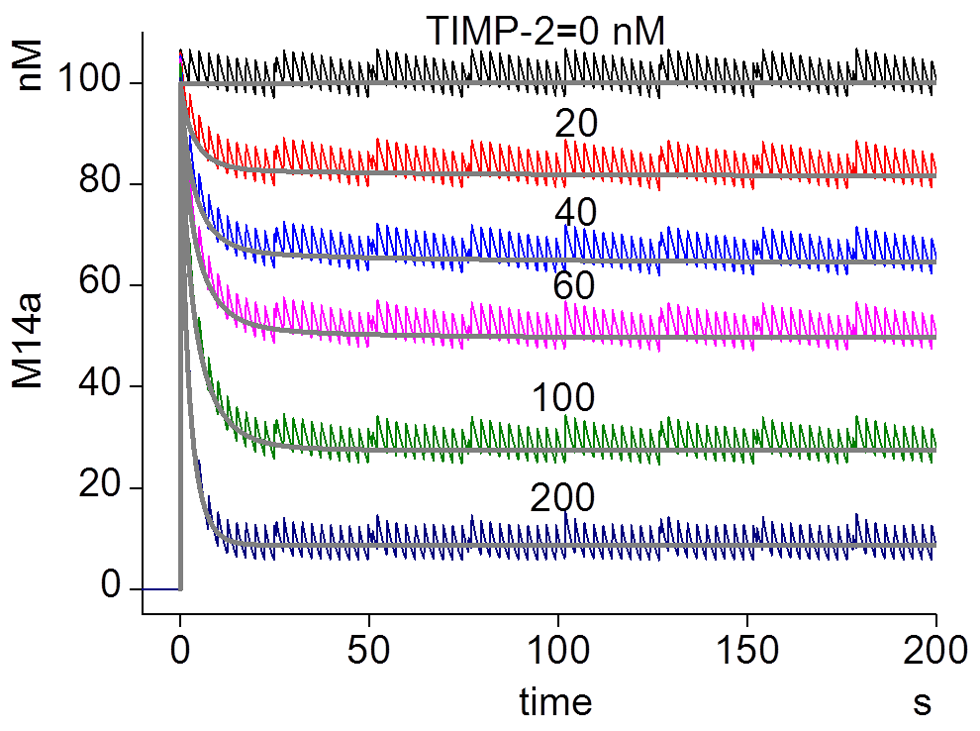

Supplement: Figure S8 — Pulsatile insertions and comparison with continuous insertion. Time courses of M14a are drawn in different colors for TIMP-2 concentration, which are overlapped by continuous insertion model in gray lines. (TIF) [file pcbi.1003086.s008.tif]

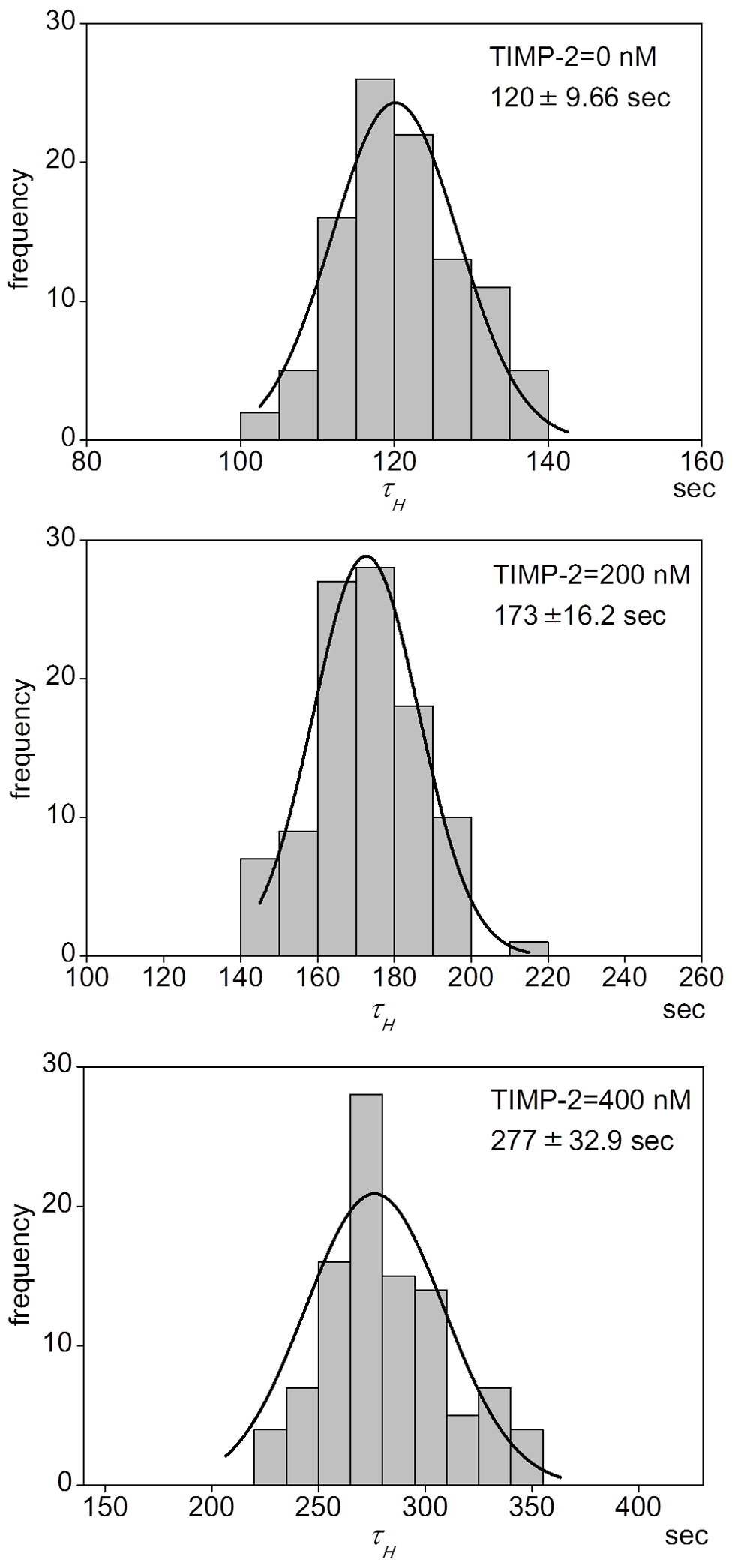

Supplement: Figure S9 — Histogram of τH for pulsatile insertion with random intervals and regular content of MT1-MMP. The average±SD of τH for degrading ECM at TIMP-2 of 0, 200, and 400 nM are 120±9.66, 173±16.2, and 277±32.9 sec, respectively. SD by pulsatile insertion is relatively small ranging from 8.05 to 11.9% of the average. (TIF) [file pcbi.1003086.s009.tif]

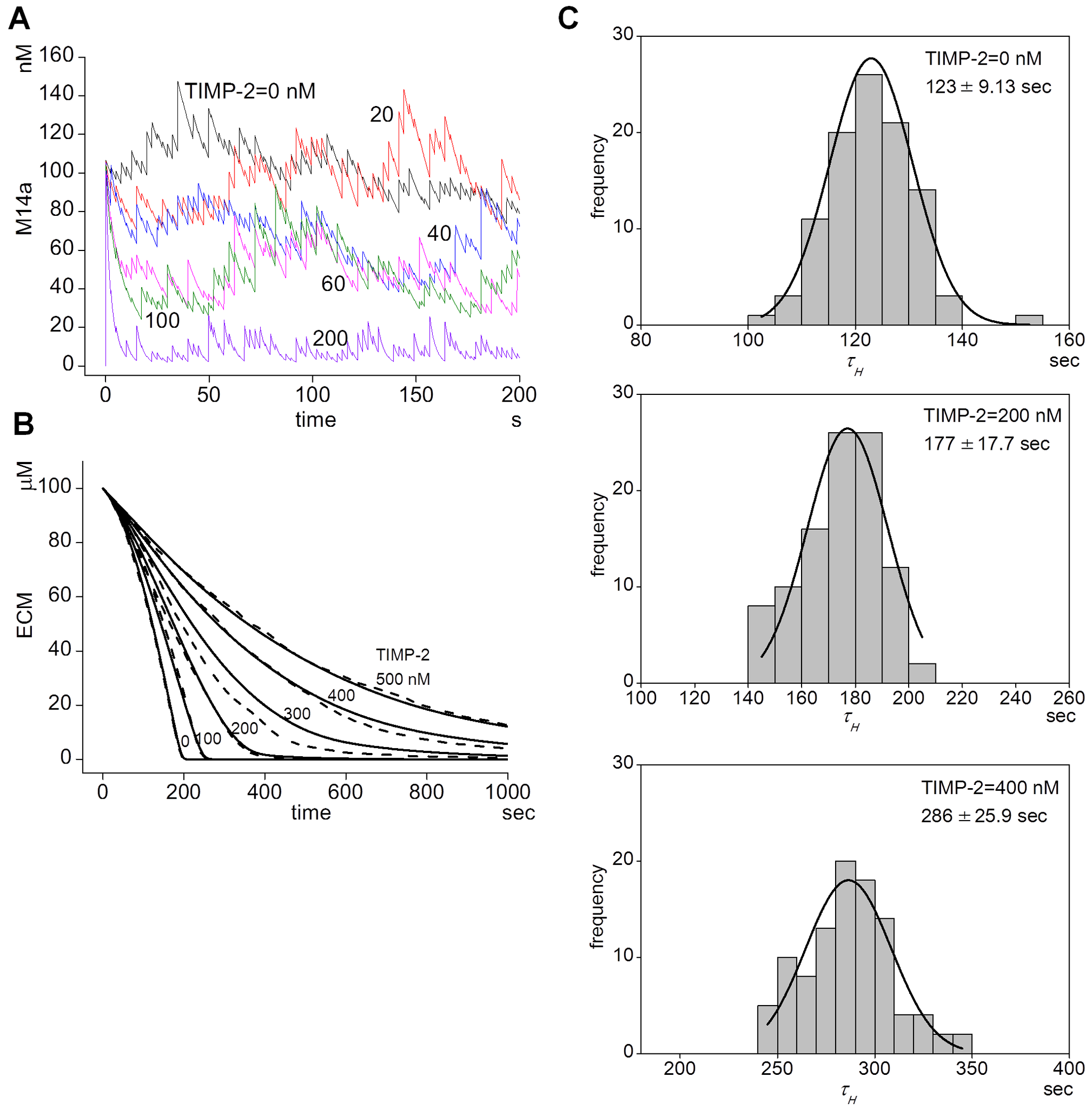

Supplement: Figure S10 — Pulsatile insertion with random amount of MT1-MMP at a single insertion. (A) The time courses of M14a for TIMP-2 from 0 to 200 nM resemble the same random behavior as in the insertion at random intervals with regular amount of MT1-MMP. (B)There are some differences in the time course of ECM degradation, but they are not large. (C) The histograms of τH show small variances as in Figure S10. (TIF) [file pcbi.1003086.s010.tif]

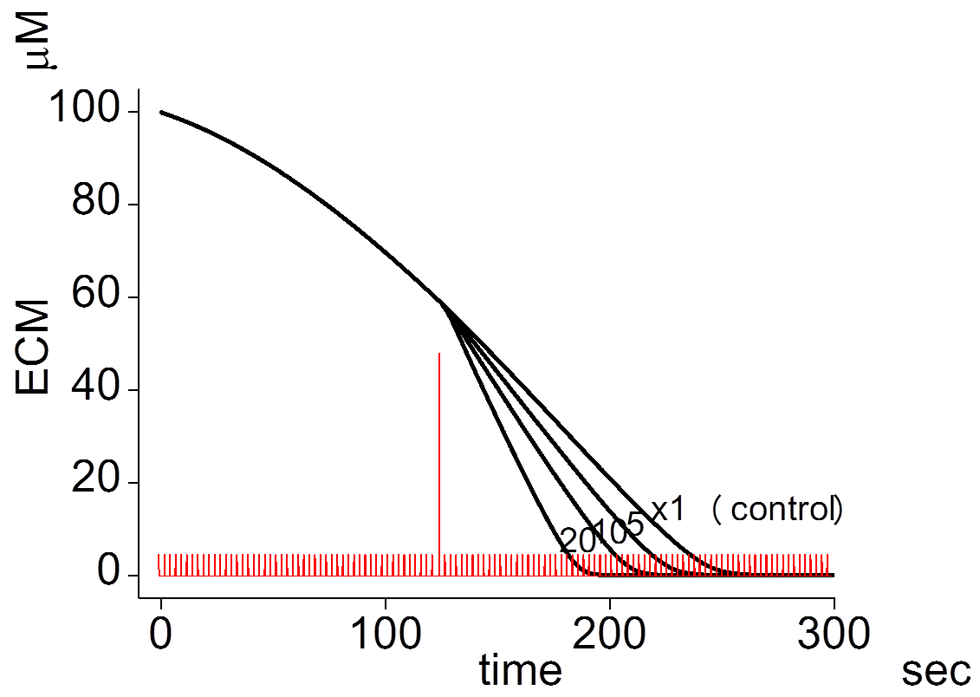

Supplement: Figure S11 — Abrupt insertion of unexpectedly large content of MT1-MMP. To see the effect of abrupt insertion event of large MT1-MMP content vesicle, we ran simulations for insertion of large MT1-MMP content at the time of τH in the course of ECM degradation as a representative event of abrupt insertion. The MT1-MMP content was 5-, 10-, 20-times of the control. (TIF) [file pcbi.1003086.s011.tif]

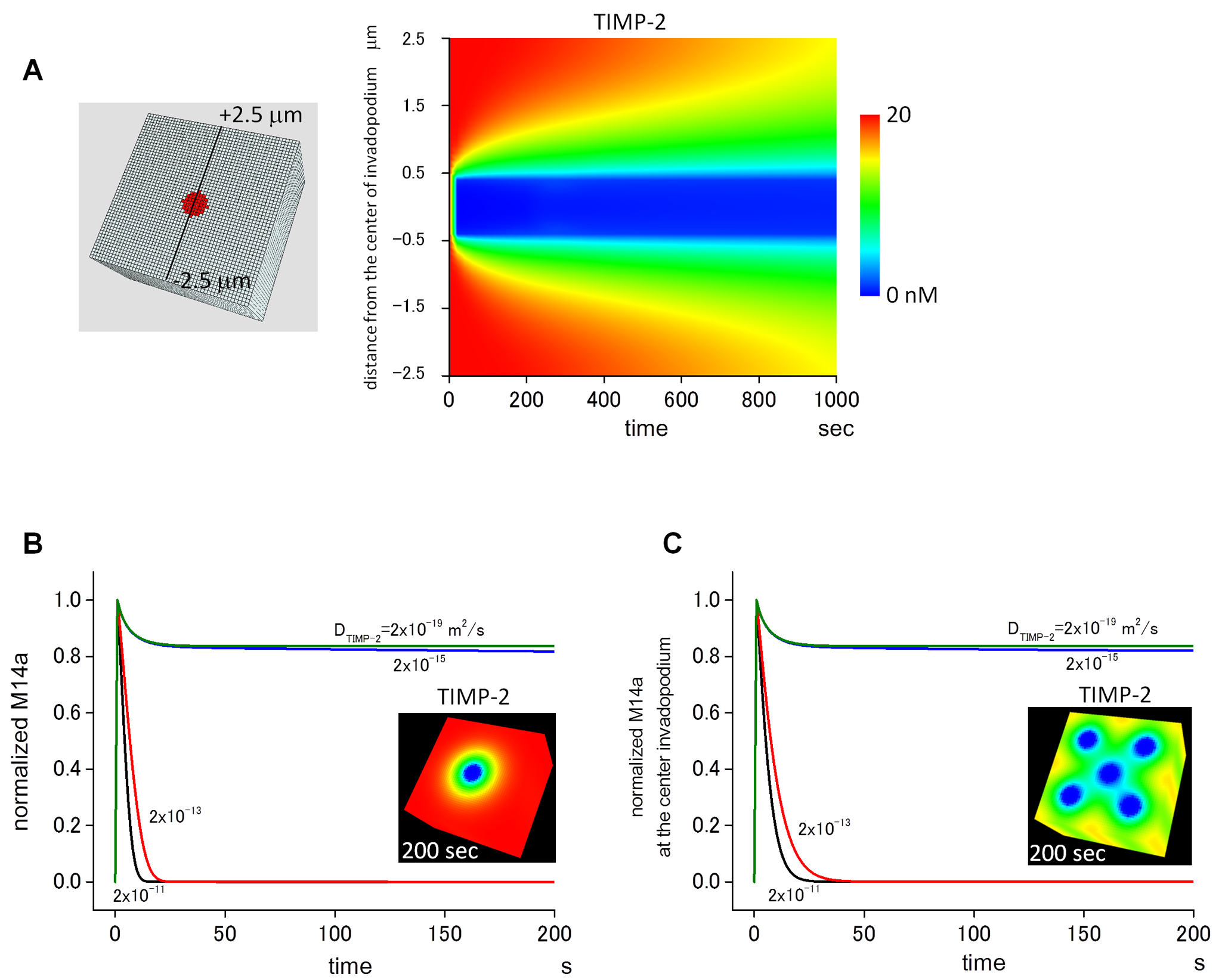

Supplement: Figure S12 — Spatio-temporal profile of TIMP-2. (A) At TIMP-2 concentration of 20 nM, there is a large spatial gradient in TIMP-2, and it grows with time. (B)(C) A sharp transient peak in MT1-MMP activity is observed at diffusion coefficient of TIMP-2 from 2×10^−19^ to 2×10^−11^ m2/s both in single invadopodium and five invadopodia simulations. Insets show spatial distribution of TIMP-2 at 200 sec. These simulation results indicate the existence of the sharp transient activity of MT1-MMP even in the limited accessibility of TIMP-2 at invadopodia. (TIF) [file pcbi.1003086.s012.tif]

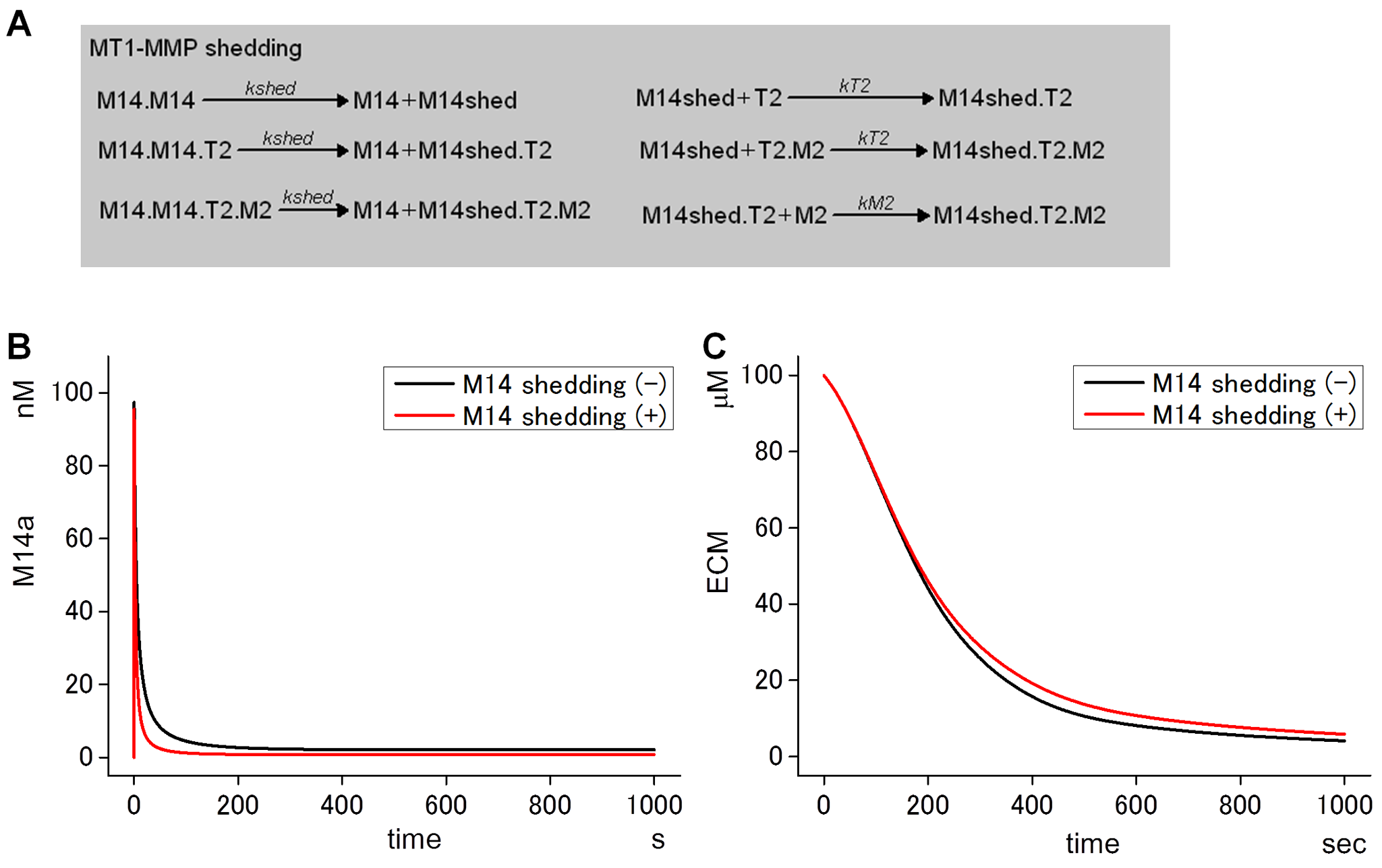

Supplement: Figure S13 — Effect of ectodomain shedding of MT1-MMP on the ECM degradation. (A) A model for the ectodomain shedding of MT1-MMP. The rate constant of shedding, kshed, is the same as used before [34]. (B) The difference in the time course of M14a in the absence and presence of the ectodomain shedding is small. (C) There is only a small difference in the time courses of ECM degradation in the absence and presence of ectodomain shedding. (TIF) [file pcbi.1003086.s013.tif]
