# Supplementary material for: Critical Role of Transient Activity of MT1-MMP for ECM Degradation in Invadopodia
Source: PLoS Comput Biol. 2013 May 30;9(5):e1003086. doi: 10.1371/journal.pcbi.1003086 (PMC3667784; doi:10.1371/journal.pcbi.1003086)
Supplement: Table S1 — Parameters for analyzing dynamics of MT1-MMP in the temporal simulations. Parameter values in this table correspond to the model shown in Figure S2. All parameter values not listed in the table are set at 0 initially. (PDF) [file pcbi.1003086.s014.pdf]

Table S1 Parameters for analyzing dynamics of MT1-MMP in the temporal simulations.

parameters	Values	unit
M14	1.00×10^{-7}	M
M2	1.00×10^{-7}	M
T2	1.00×10^{-7}	M
kM14	2.00×10^{6}	/M/s
k_M14	0.01	/s
kM2	2.10×10^{7}	/M/s
kT2	2.74×10^{6}	/M/s
k_T2	2.00×10^{-4}	/s
