# Supplementary material for: Critical Role of Transient Activity of MT1-MMP for ECM Degradation in Invadopodia
Source: PLoS Comput Biol. 2013 May 30;9(5):e1003086. doi: 10.1371/journal.pcbi.1003086 (PMC3667784; doi:10.1371/journal.pcbi.1003086)
Supplement: Table S2 — Parameters for analyzing dynamics of MT1-MMP with pools X and D in the temporal simulations. Parameter values in this table correspond to the model shown in Figure S6. All parameter values not listed in the table are set at 0 initially. (PDF) [file pcbi.1003086.s015.pdf]

Table S2 Parameters for analyzing dynamics of MT1-MMP with pools X and D in the temporal simulations.

parameters	Values	unit
Cpd・CD	$2.69x10^{-9}$	/s
CX	$1.18x10^{-10}$	M/s
M14D	$6.99x10^{-8}$	M
M14x	$3.01x10^{-8}$	M
M2	$1.00x10^{-7}$	M
MF	$3.06x10^{-8}$	M
Ms	$6.06x10^{-8}$	M
T2	$1.00x10^{-7}$	M
kD	0.0385	/s
kM14	$2.00x10^{6}$	/M/s
k_M14	0.01	/s
kM2	$2.10x10^{7}$	/M/s
kT2	$2.74x10^{6}$	/M/s
k_T2	$2.00x10^{-4}$	/s
kX	0.00386	/s
